# Electrospun fiber‐based immune engineering in regenerative medicine

**DOI:** 10.1002/SMMD.20230034

**Published:** 2024-02-24

**Authors:** Yiru Xu, Qimanguli Saiding, Xue Zhou, Juan Wang, Wenguo Cui, Xinliang Chen

**Affiliations:** ^1^ The International Peace Maternity and Child Health Hospital School of Medicine Shanghai Jiao Tong University Shanghai China; ^2^ Shanghai Key Laboratory of Embryo Original Diseases Shanghai China; ^3^ Department of Orthopaedics Shanghai Key Laboratory for Prevention and Treatment of Bone and Joint Diseases Shanghai Institute of Traumatology and Orthopaedics Ruijin Hospital Shanghai Jiao Tong University School of Medicine Shanghai China

**Keywords:** electrospinning, immune engineering, macrophages, regenerative medicine, tissue repair

## Abstract

Immune engineering, a burgeoning field within regenerative medicine, involves a spectrum of strategies to optimize the intricate interplay between tissue regenerative biomaterials and the host tissue. These strategies are applied across different types of biomaterials and various disease models, which encompasses finely modulating the immune response at the levels of immune cells and factors, aiming to mitigate adverse effects like fibrosis and persistent inflammation that may arise at the injury site and consequently promote tissue regeneration. With the continuous progress in electrospinning technology, the immunoregulatory capabilities of electrospun fibers have gained substantial attention over the years. Electrospun fibers, with their extracellular matrix‐like characteristics, high surface‐area‐to‐volume ratio, and reliable pharmaceutical compound capacity, have emerged as key players among tissue engineering materials. This review specifically focuses on the role of electrospun fiber‐based immune engineering, emphasizing their unique design strategies. Notably, electrospinning actively engages in immune engineering by modulating immune responses through four essential strategies: (i) surface modification, (ii) drug loading, (iii) physicochemical parameters, and (iv) biological grafting. This review presents a comprehensive overview of the intricate mechanisms of the immune system in injured tissues while unveiling the key strategies adopted by electrospun fibers to orchestrate immune regulation. Furthermore, the review explores the current developmental trends and limitations concerning the immunoregulatory function of electrospun fibers, aiming to drive the advancements in electrospun fiber‐based immune engineering to its full potential.

## Graphical abstract

Specially engineered electrospun fibers can modulate the immune cells at the implantation site, such as macrophages and neutrophils, inducing them to adopt an anti‐inflammatory cell phenotype or secrete anti‐inflammatory cytokines, thereby exerting immunomodulatory effects.

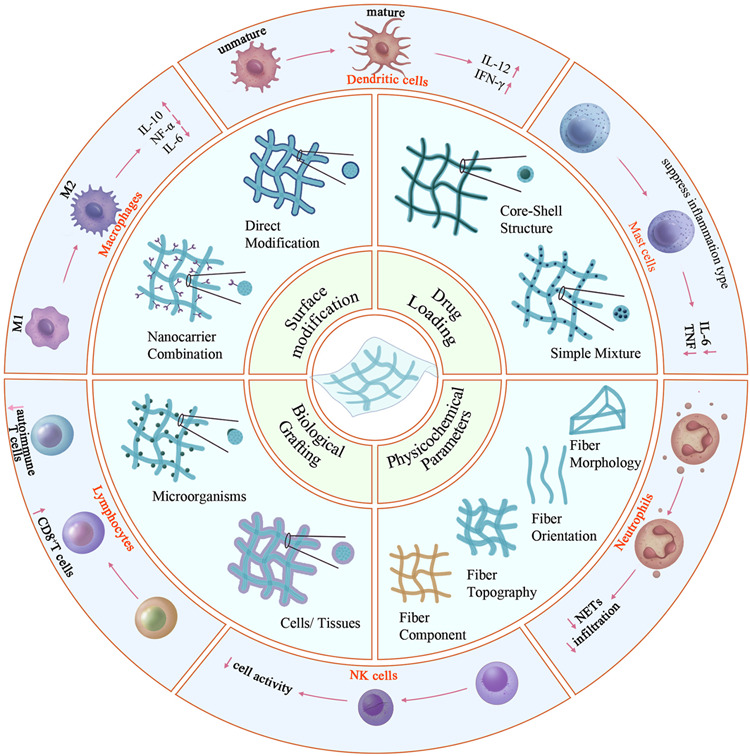

**Key points**

- Electrospun fiber techniques in immune engineering to uniquely modulate the organ's immune response.
- Latest advancements in immunomodulatory electrospun fibers.
- Using electrospinning for immune modulation and its upcoming directions.
- The use and hurdles of immunomodulatory electrospun fibers in the realm of regenerative medicine.

## 1 INTRODUCTION

In the past few years, tissue engineering materials have undergone a remarkable expansion and have been widely utilized across various medical disciplines.^1^, ^2^ These materials serve multiple purposes in medical applications, such as assisting in wound healing,^3^, ^4^ replacing blood vessels and heart valves,^5^, ^6^, ^7^ reconstructing pelvic organ prolapse^8^ and repairing abdominal wall hernias,^9^ promoting bone and nerve regeneration,^10^ and even acting as therapeutic agents in tumor treatments.^11^ Tissue engineering materials come in various forms, including electrospun fibers,^12^ hydrogels,^13^ microspheres,^14^ liposomes,^8^, ^15^ and nanoparticles.^16^ Investigative studies have unveiled through thoughtful design, that these biomaterials possess the ability to modulate local immune responses, promoting tissue healing and facilitating functional restoration.^17^ Notwithstanding their promising potential, the foreign nature of these materials often induces host immune reactions, leading to various complications, including mechanical encapsulation, chronic sterile inflammation, infections, and recurrences.^18^, ^19^ Thus, the critical task of steering the local environment toward an anti‐inflammatory trajectory and mitigating the host immune response to biomaterials remains an inherent challenge within tissue engineering materials.

The immune system operates with precise control and balance. Under optimal physiological conditions, the immune system serves as a formidable defense mechanism against external stimuli, safeguarding the internal milieu and maintaining homeostasis. However, its dysregulation is central to various diseases.^20^ The immune system comprises innate and adaptive immunity, exerting its effects through diverse immune cells and cytokines.^21^ Intense immune cells involve macrophages, neutrophils, dendritic cells (DCs), mast cells, and natural killer cells (NK). In contrast, the adaptive immune cells include T and B cells among other types of cells.^22^ These immune cells are recruited and activated upon encountering pathogens, foreign substances, and other stimuli, enabling the presentation of antigens and the secretion of inflammatory mediators such as interleukin‐1 (IL‐1), IL‐6, tumor necrosis factor‐α (TNF‐α), as well as anti‐inflammatory factors like IL‐4 and IL‐10.

A complex and intricate interplay exists between biomaterials and the immune system. Upon implantation, adhesive proteins, including fibrinogen, fibronectin, albumin, immunoglobulins, and complement proteins, rapidly adhere to the surface of biomaterials, giving rise to a protein layer.^23^ These proteins are then recognized by antigen‐presenting cells (APCs) such as DCs, triggering subsequent host immune responses.^24^ During the early stages, neutrophils play a crucial role by engulfing dead cells and attracting inflammatory cells to the affected area.^25^ Over time, macrophages gradually infiltrate and become the predominant cell population, exhibiting both proinflammatory and anti‐inflammatory phenotypes and eventually determining the outcome through the extent and speed of their phenotypic transition.^26^ Mast cells and lymphocytes also contribute to the response to varying degrees.^27^

Beyond the host immune response elicited by material implantation, the implantation site often displays aberrant immune responses, eventually ending in impaired tissue regeneration, consequently necessitating the exertion of immunomodulatory effects by materials to facilitate regeneration.^28^ For instance, tumor progression results from immune evasion and the tumor microenvironment, characterized by acidity and increased levels of reactive oxygen species (ROS).^29^ Materials utilized for applications in the context of tumors are expected to manifest the capacity to modulate ROS, thereby impeding the continued proliferation of tumors.^30^ In addition, excessive or inappropriate immune reactions will impede wound healing or lead to fibrosis.^31^ Materials applied to persistent cutaneous wounds are envisioned to possess the capability to regulate enduring inflammatory responses, thereby fostering the wounds while suppressing the incidence of infection and the development of scars.^32^ Hence, biomaterials must minimize their inherent immunogenicity and prioritize their immunomodulatory capabilities to prevent secondary harm to the body and the potential persistence of chronic inflammatory reactions, ultimately promoting the restoration of homeostasis of the target organ.

Nowadays, among the thriving progress in regenerative medicine, immune engineering strategies have garnered widespread adoption. These methodologies serve as the fundamental design principles in the conception of biomaterials. The goal of implementing immune engineering strategies is to govern the interplay between implanted biomaterials and the host's immune response, steering it toward facilitating regeneration.^33^ This endeavor aims to prevent the occurrence of intense rejection reactions and alleviate potential inflammation at the implantation site. The concentration extends to the realm of immune cells, immune factors, and even the modulation of signaling pathways all to attain the immune regulatory functionalities inherent in the designed materials.^34^

**FIGURE 1:**
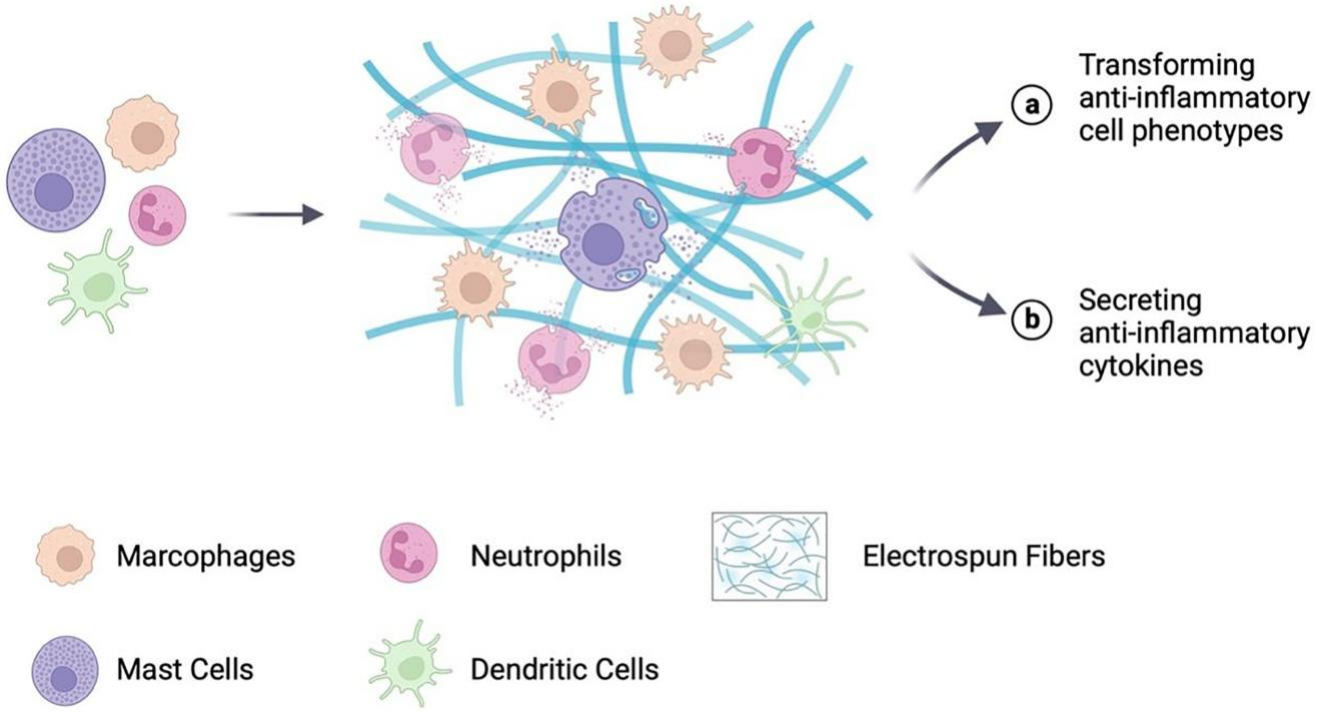
The immunoregulatory potential of electrospun fibers during the tissue repair process. Created with BioRender.com.

**FIGURE 2:**
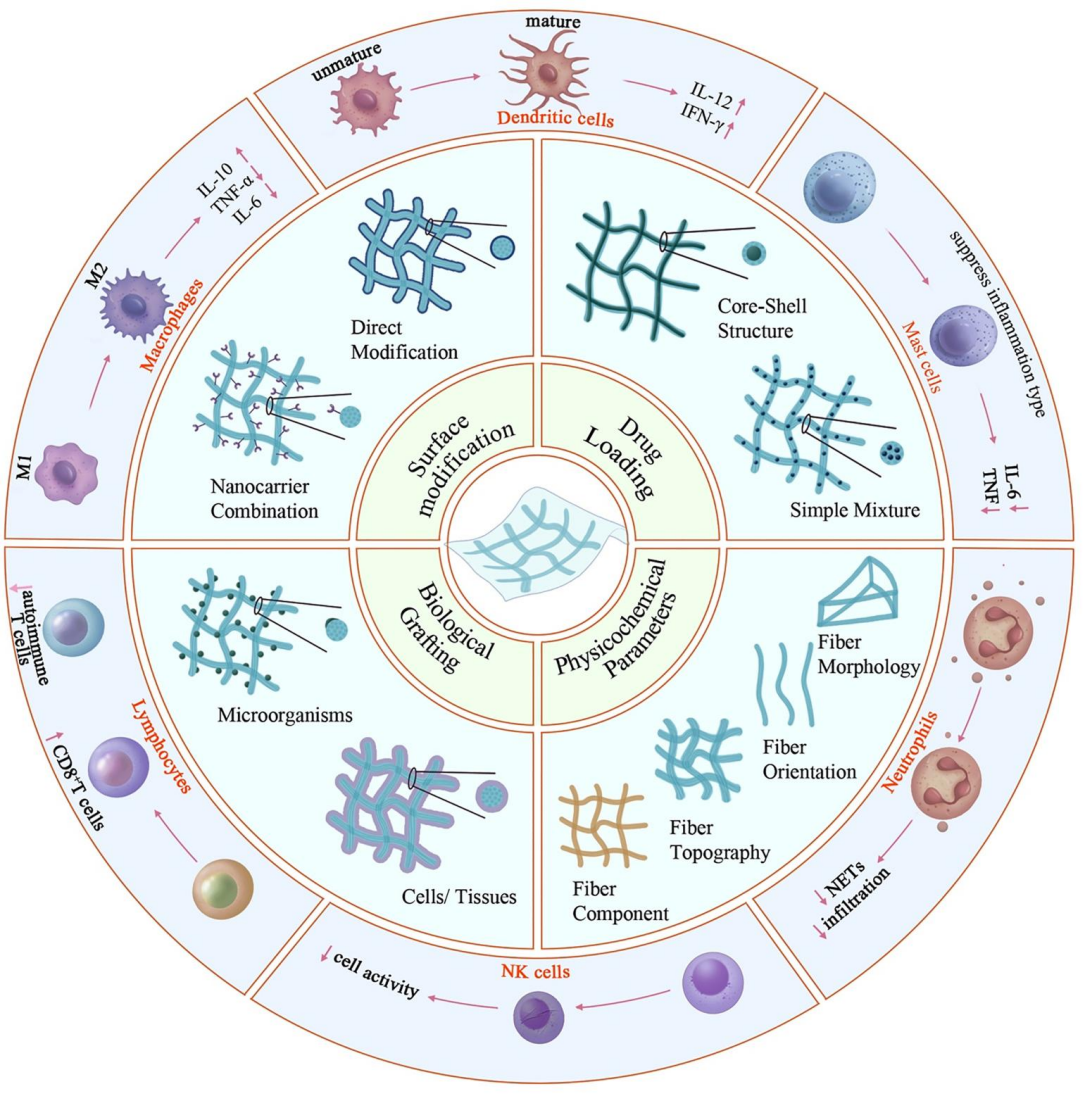
Schematic illustration of electrospun fiber‐based immune engineering.

Among the multitude of biomaterials, electrospun fibers stand out due to their applicability in immune engineering strategies, attributed to advantages such as a large surface‐to‐volume ratio, high porosity, extracellular matrix (ECM)‐like three‐dimensional (3D) structure, exceptional biocompatibility, and favorable drug loading capacity.^35^, ^36^, ^37^, ^38^ For instance, after applying immune engineering strategies, they can achieve immune modulation through macrophage polarization and neutrophil aggregation, highlighting their impressive immunoregulatory abilities (Figure 1).^39^, ^40^ Nevertheless, a comprehensive overview elucidating the impact of electrospinning immune engineering on immune cells and summarizing the strategies employed to modulate immune processes is presently absent. To update the present knowledge regarding electrospun fiber‐based immune engineering in regenerative medicine, this review summarizes the immune regulative strategies within the scope of various immune cells and fiber designs, including surface modification, drug loading, physicochemical parameters, and biological grafting that are intended to steer immune directions favorable to tissue regeneration (Figure 2). This work aims to offer valuable insights for the future design of electrospun‐based immune engineering materials with current challenges and promising perspectives.

## 2 ELECTROSPUN FIBER‐BASED STRATEGIES TO REGULATE IMMUNE CELLS

Due to the pivotal role played by the degree of activation in immune cells in determining the direction of immune responses, the modulation of immune cells is a crucial objective in designing electrospinning strategies in immunological engineering. Typical immune cells encompass macrophages, DCs, neutrophils, lymphocytes, NK cells, and tissue cells. Electrospinning in immunological engineering has been applied to regulate the activation levels of specific immune cells and has found application in various disease models.

### 2.1 Macrophages

In the immune system, macrophages serve as pivotal players, acting dually as antimicrobial effectors and as architects of immunomodulation. Their capacity to elicit, suppress, or finely tune adaptive immune responses renders them influential judges in determining the fate of implanted biomaterials and the efficacy of tissue regeneration.^41^ Considering the paramount significance of macrophages within the immune system, numerous immunomodulatory strategies employing electrospinning endeavor to enhance immune regulation through the modulation of macrophage phenotypes.^42^

**FIGURE 3:**
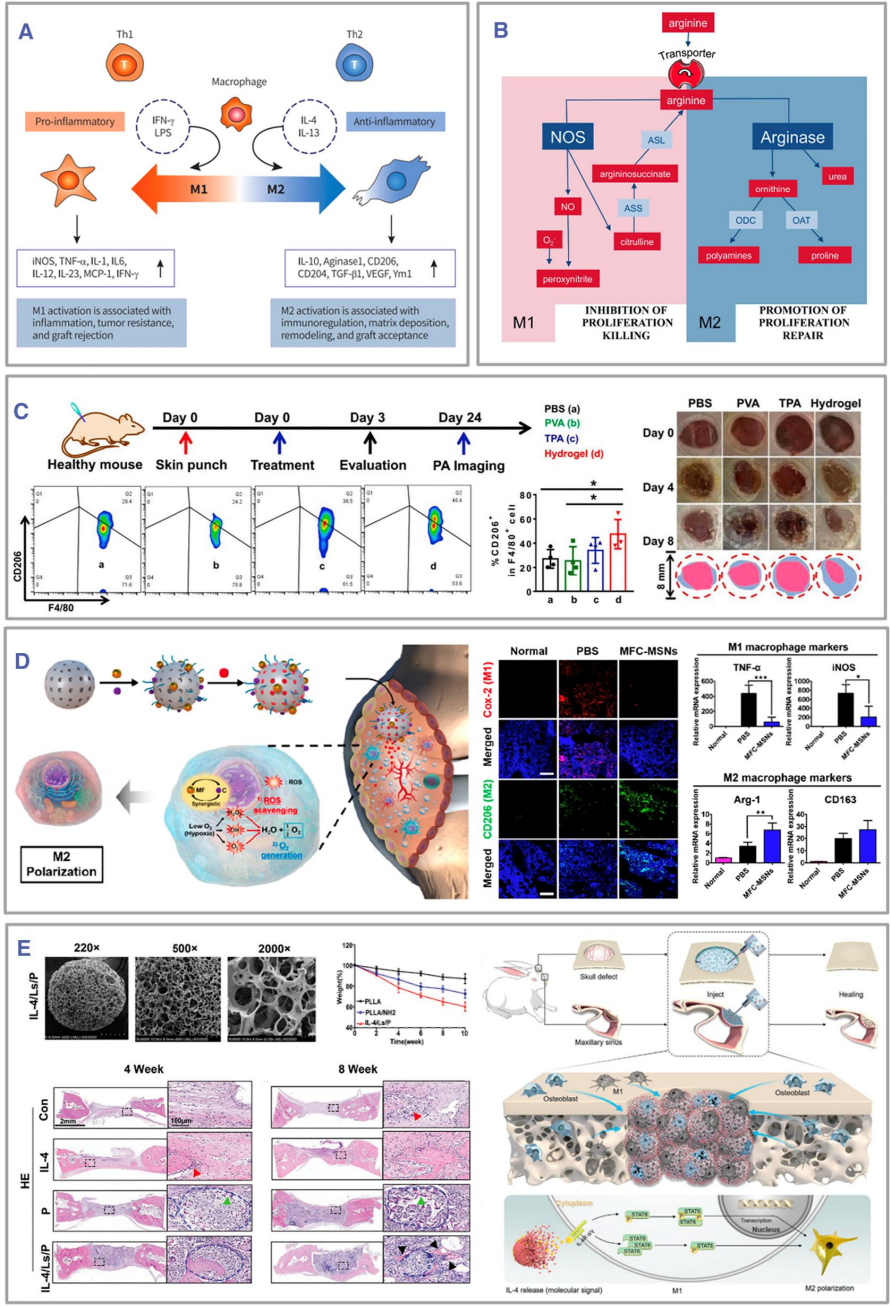
Macrophage polarization by various immune engineering strategies. (A) M1 and M2 macrophages express distinctive surface markers and produce diverse cytokines with specific tissue repair outcomes. Reproduced with permission.^43^ Copyright 2019, Medical Biological Science and Engineering. (B) Metabolic pathway modulation for macrophage polarization. Reproduced under terms of the CC‐BY license.^44^ Copyright 2014, The Authors, published by Frontiers Media S. A. (C) Macrophage polarization by hydrogels. *p < 0.05. Reproduced with permission.^45^ Copyright 2020, Elsevier Ltd. (D) Macrophage polarization by nanoparticles. Scale bars: 100 μm. *p < 0.05, **p < 0.01 and ***p < 0.001. Reproduced with permission.^46^ Copyright 2019, American Chemical Society. (E) Macrophage polarization by microspheres. Reproduced with permission.^47^ Copyright 2022, John Wiley and Sons.

The macrophages are generally classified into M0, M1, and M2. Once activated, M0 macrophages can polarize into M1 or M2 phenotypes.^43^ The divergent polarization of M1 and M2 gives rise to contrasting inflammatory reactions, with M1 exhibiting a proinflammatory nature and M2 displaying anti‐inflammatory characteristics (Figure 3A). M1 macrophages denote the classically activated phenotype possessing robust abilities in antigen presentation. They are typically induced by Th1 cytokines (such as interferon‐gamma [IFN‐γ] and TNF‐α) or microbial lipopolysaccharides, leading to enhanced expression of proinflammatory factors (such as TNF‐α, IL‐1α, IL‐1β, IL‐6, IL‐12, IL‐23) and chemokines (such as chemokine (C‐X‐C motif) ligand 9 [CXCL9], CXCL10, and CXCL11), while demonstrating lower levels of IL‐10. Surface markers include the cluster of differentiation (CD) 86 and inducible nitric oxide synthase (iNOS). Conversely, M2 macrophages are immunoregulatory phagocytes with anti‐inflammatory properties triggered by T helper 2 (Th2) cytokines (such as IL‐4 and IL‐13), IL‐10, IL‐33, IL‐21, and the beyond. They manifest elevated expression of IL‐10 and transforming growth factor‐beta (TGF‐β), reduced expression of IL‐12, and surface markers like CD206 and CD204. M2 macrophages can be subdivided into M2a, M2b, and M2c subtypes, with M2a identified as alternatively activated macrophages, M2b as immunoregulatory macrophages, and M2c recognized as deactivating macrophages engaged in mitigating inflammation and facilitating tissue repair.^48^

Macrophages exhibit different phenotypes due to variations in their arginine metabolic pathways (Figure 3B).^44^ M1 macrophages express iNOS which catalyzes the conversion of arginine into nitric oxide (NO) and citrulline. NO can subsequently undergo metabolic transformations into downstream reactive nitrogen species, driving the progression of inflammation and contributing to neovascularization.^49^ On the other hand, citrulline can be reused through the citrulline–NO cycle to support NO synthesis. In contrast, M2 macrophages express arginase, an enzyme that can hydrolyze arginine into ornithine and urea. The arginase pathway limits arginine availability for NO synthesis, while ornithine is a precursor for synthesizing polyamines and proline, facilitating cellular proliferation and tissue repair. Notably, the metabolic byproducts of these pathways exhibit reciprocal inhibitory effects and can influence the differentiation of T helper cells toward the Th1/Th2 types. In turn, the differentiated Th cells further enhance the polarization of the macrophages.^50^

Besides, the types of macrophages that predominate vary for different diseases. For instance, in the context of osteoarthritis, synovial macrophages transform into the M1 phenotype, governed by signaling pathways encompassing the mammalian target of rapamycin (mTOR), nuclear factor‐kappa B (NF‐κB), c‐Jun N‐terminal kinase, and phosphatidylinositol 3‐kinase/protein kinase B (PI3K/Akt).^51^ Conversely, in Alveolar Echinococcosis, *Echinococcus multilocularis protoscoleces* promote M2 polarization of macrophages, thereby contributing to the genesis of hepatic cysts by activating the PI3K/Akt/mTOR signaling pathway.^52^

However, the phenotypic manifestation of macrophages is not rigid; under suitable external stimuli, diverse phenotypes can interconvert. Infections, signals indicative of acute damage, and implanted biomaterials can impact this process by triggering many signaling pathways. The ability of macrophages to modulate the attributes of their phenotype through diverse signaling pathways forms a crucial focal point in immunomodulation strategies based on electrospinning.^53^ Adequate stimulation agitates signaling pathways such as signal transducers and activators of transcription,^54^ Toll‐like receptor 4 (TLR4)/IL10,^55^ and NF‐κB/mitogen‐activated protein kinases,^56^ fostering the interconversion of phenotypes and playing a role in the pathogenesis of diverse ailments.

The utilization of biomaterials in diverse disease models seeks to facilitate regeneration, where the dysregulation of macrophage phenotypes frequently acts as the cause or consequence of disease advancement. Thus, within biocompatible material applications, an imbalance in inflammatory reactions often manifests or emerges at the implantation site. For instance, biocompatible materials can function as wound dressings, where macrophages at a typical wound site predominantly adopt an M2 phenotype 3 days post‐injury. However, diabetic wounds often demonstrate disrupted and enduring M1 macrophage polarization, resulting in delayed wound healing.^57^

Therefore, biomaterials must be able to modulate macrophage polarization to regulate local immunity, avoid inflammatory dysregulation, and promote tissue regeneration.^58^ For example, polyvinyl alcohol (PVA) can be cross‐linked with N1‐(4‐boronobenzyl)‐N3‐(4‐boronophenyl)‐N1, N1, N3, N3‐tetramethylpropane‐1, 3‐diaminium (TPA), a ROS‐responsive linker, to form ROS‐scavenging hydrogels that promote M2 macrophage transitioning and wound healing (Figure 3C).^45^ Ferrite and ceria nanoparticle‐anchored mesoporous silica nanoparticles can scavenge ROS and promote M2 polarization and have been used in treating rheumatoid arthritis (Figure 3D).^46^ Moreover, IL‐4‐loaded liposomes/poly(l‐lactic acid) (IL‐4/Ls/PLLA) microspheres were obtained via the amide bond modification of IL‐4‐containing liposomes on PLLA microspheres, which can induce M2 polarization and promote bone regeneration (Figure 3E).^47^ However, these materials are susceptible to degradation and cannot provide mechanical support for damaged tissues.

**FIGURE 4:**
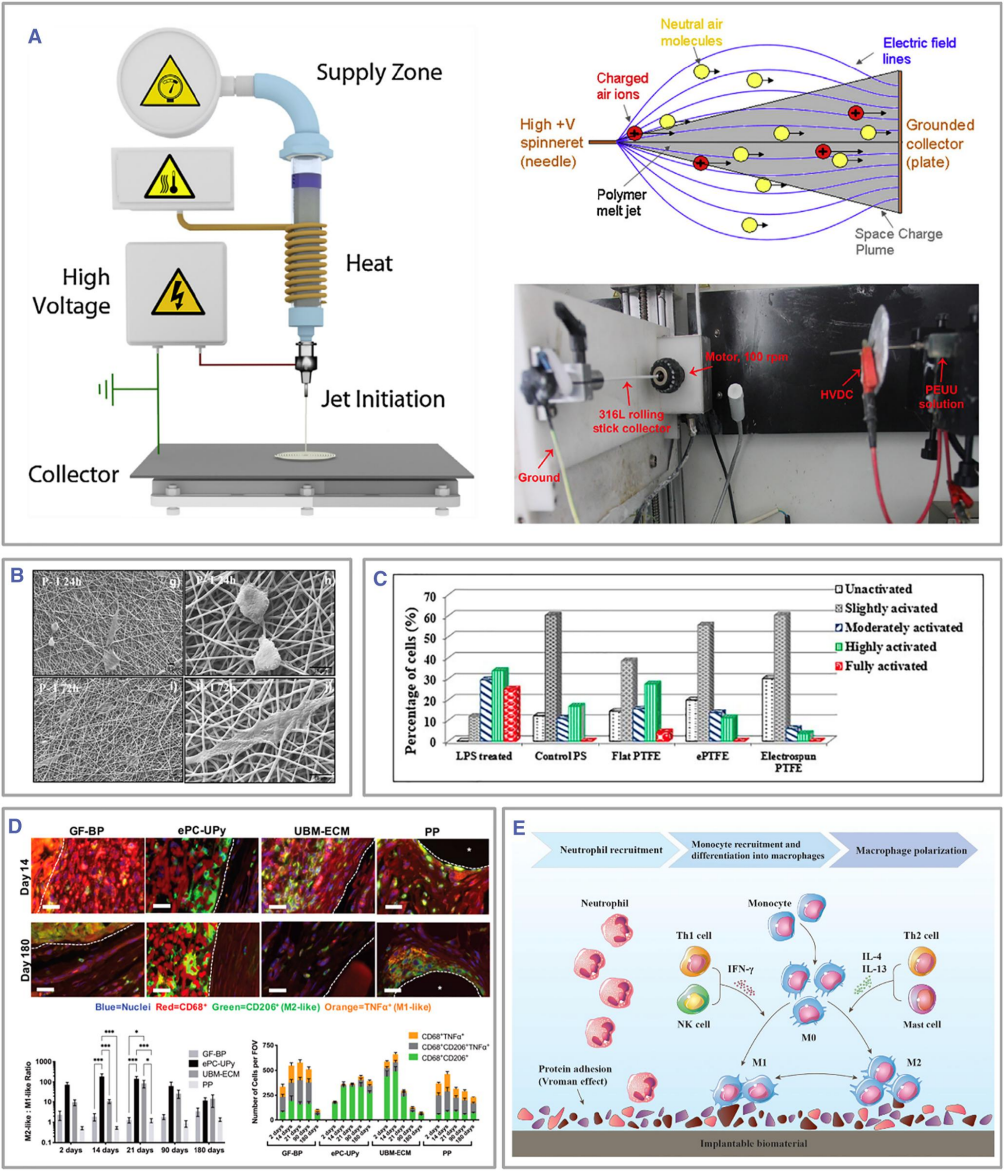
(A) Schematic illustration of electrospinning device, mechanism, and process. Reproduced with permission.^60^, ^61^ Copyright 2019, American Chemical Society; Copyright 2021, Elsevier. (B) SEM images of an M2‐polarized phenotype after 72 h. Reproduced with permission.^62^ Copyright 2021, Elsevier. (C) PTFE electrospun fibers obtained by electrospinning show less agitation of macrophages after 24 h of co‐culture. Reproduced with permission.^63^ Copyright 2017, John Wiley and Sons. (D) Electrospun fibers implanted in vivo elicit varied macrophage phenotypes depending on material type and implantation duration. Scale bars: 25 μm. Reproduced with permission.^64^ Copyright 2021, John Wiley and Sons. (E) Scheme of the immune response triggered by the implantation of biomaterials in the body. *p < 0.05 and ***p < 0.001. Reproduced under terms of the CC‐BY license.^24^ Copyright 2021, The Authors, published by Frontiers Media S. A. PTFE, polytetrafluoroethylene.

Electrospun fibers exhibit a more pronounced capacity for inducing macrophage polarization than other biomaterial forms.^59^ Electrospinning represents a highly versatile and viable technique for fabricating ultrathin fibers.^60^ Its essential constituents encompass a high‐voltage power supply, an infusion pump, a spinneret, and a conductive collector (Figure 4A).^61^ During the electrospinning process, the liquid is extruded from the spinneret, forming droplets under the influence of surface tension. Upon electrification, electrostatic repulsion between like‐signed surface charges induces droplet deformation into a Taylor cone, generating charged jets that ultimately solidify into fibers on a grounded collector, commonly referred to as electrospun fibers.^65^, ^66^ Such fibers exhibit ECM‐like attributes and demonstrate exceptional biocompatibility.^67^ M0 macrophages adhered to poly‐L‐lactide (PLA): poly‐ε‐caprolactone (PCL) = 7:3 electrospun fibers manifest elongation and an upregulated expression of IL‐10, indicative of M2 polarization at the 72 h mark (Figure 4B).^62^ What's more, contrasting flat polytetrafluoroethylene (PTFE) and expanded PTFE, electrospun PTFE evokes diminished macrophage activation and fewer foreign body giant cells (Figure 4C).^63^ When juxtaposed with clinically utilized materials like polypropylene mesh, glutaraldehyde‐treated bovine pericardium, and porcine bladder ECM (UBM‐ECM), polycarbonate electrospun fibers offer advantages, including a higher M2:M1 ratio, reduced propensity for calcification, and facilitation of neovascularization (Figure 4D).^64^

In addition, macrophages harbor the ability to engulf extraneous entities, positioning a pivotal role as the predominant cellular mediator in host rejection responses, thereby substantially impacting the tissue repair outcomes of implanted electrospun fibers.^68^ After implantation, an array of adhesive proteins adsorbs onto the surface, conferring its identification as a foreign entity by the organism, thereby promptly recruiting and activating macrophages. During the initial phase, macrophages predominantly exhibit an M1 phenotype, releasing multiple inflammatory mediators that incite localized acute inflammation. Ideally, macrophages should transit to an M2 phenotype after this acute phase, facilitating inflammation resolution and tissue healing (Figure 4E).^24^ Macrophages are the most vital cells mediating the degradation of electrospun fibers.^69^ If the implanted electrospun fibers are non‐degradable and the macrophages do not show a tendency toward M2 polarization, local chronic inflammation will persist, and the macrophages will fuse into foreign‐body giant cells, causing fibroblast recruitment, excessive collagen deposition, and material encapsulation to form fibrous capsules, contrary to tissue healing progress.^70^

Thus, owing to the pivotal role of macrophages within the immune system and recognizing that implanted electrospun fibers can intricately regulate the phenotype of macrophages, it is imperative in the orchestration of electrospinning to contemplate the predominant macrophage types prevailing in the targeted diseases. Beyond mitigating innate host rejection responses, the objective is to nurture a balance of macrophage phenotypes at the implantation site. Among diverse electrospun fiber‐based immune engineering strategies directed at a multitude of immune cells, strategies centering on the modulation of macrophage phenotypes were the earliest to surface and presently stand as the most extensively applied. Electrospinning conceived through the prism of these immune engineering strategies finds versatile applications across a myriad of disease scenarios.^71^, ^72^ Studies have shown that these approaches can enhance M2 polarization and promote tissue regeneration, which has been demonstrated in applications such as skin, nerve, and bone repair as well as angiogenesis at both the cellular and organ levels (Table 1). Moreover, electrospun fibers possess inherent design flexibility, enabling them to augment macrophage polarization by altering the fibers' physicochemical parameters, performing surface modifications, incorporating anti‐inflammatory substances or modifying surface topographical structures (see Tables 3 and 4).

**TABLE 1:** Applications of electrospun fibers for regulating macrophage polarization.

Polymer type	Electrospun fibers design	Application site	Macrophage polarization	Effect on cytokines	Verification method	Refs
PCL	Macroporous, non‐woven, and heat treated	Peripheral nerves	Yes	TGF‐β^−^, IL‐1^−^, IL‐10^+^	IHC, qPCR, HE	73
PLA	Core‐shell structure, cationic liposome containing IL‐4 plasmids on the surface, core containing NGF	Spinal cord	Yes	IL‐1^−^, TNF‐α^−^, IL‐10^+^, TGF‐β^+^	FCM	74
PLCL	Grafting eMSC	Pelvic floor	Yes	IL‐6^−^, TNF‐α^−^	HE, IHC, qPCR	75
PCL	Lading aspirin‐triggered resolvin D1	Vascular	Yes	TNF‐α^−^, CXCL1^−^	qPCR, WB, IHC	76
PLLA	Layer‐by‐layer structure (3D printing PCL and electrospun PLLA)	Bone	Yes	IL‐4^+^, IL‐10^+^, IL‐6^−^, TNF‐α^−^	qPCR, FCM, IHC	77
PLA	“Inner‐outer” structure, containing 10 μg/mL HA + IL‐10	Skin	Yes	IL‐10^+^	FCM, HE, IHC	78

Abbreviations: eMSC, endometrial mesenchymal stem cell; FCM, flow cytometry; HA, hyaluronic acid; HE, hematoxylin‐eosin staining; IHC, immunohistochemistry; IL, interleukin; NGF, nerve growth factor; PCL, poly‐ε‐caprolactone; PLA, poly‐L‐lactide; PLCL, Poly(L‐lactide‐co‐caprolactone); qPCR, real‐time quantitative polymerase chain reaction; TGF‐β, transforming growth factor‐beta; WB, western blot.

Immunity and regeneration of the body is a complex system where, in addition to macrophages, other immune cells—such as DCs, mast cells, neutrophils, lymphocytes, and NK cells—play essential roles in local immune regulation during tissue repair.^79^ Electrospun fibers can also modulate these cells to relieve inflammation and restore the homeostatic state of an organism (Table 2).

**TABLE 2:** Applications of electrospun fibers in regulating other immune cells.

Polymer type	Application site/disease	Electrospun fibers design	Type of immune response	Type of immune cells	Experimental method	Refs
PCL	Inflammation	Surface modified with peptides	Host immunity	Neutrophils	Fluorescence	80
PLLA	Breast cancer	Surface modified with polydopamine and CD40mAb	Antitumor immunity	DCs	FCM	81
PVP	Subcutaneous tumors	Surface modified with microspheres containing IL‐12 plasmids	Antitumor immunity	DCs	IHC, FCM, CCK8	82
PVA	Wound repair	Loading chitosan, ox‐carbon nano‐onions	Inflammatory response	Lymphocytes, etc	HE	83
PDO	Revascularization	Surface modified with fibronectin	Host immune response	Mast cells	ELISA	84
PCL	Surgical wound excipients	Loading 1,25(OH)2D3	Bacterial infection	Monocytes/white blood cells	IHC, CCK8	85
PEOT/PBT	Tympanitis	Surface modified with CNs	Inflammatory response	Epidermal cells	qPCR	86
PVP	Tumors	Surface modified with rabbit hemorrhagic fever virus (VLP)	Innate immunity	T cells, DCs	ELISA, T‐cell proliferation assay	87
PVP	Oral lichen planus	Loading clobetasol‐17‐propionate	Autoimmune response	T cells	ELISA	88
PCL	Breast cancer	Umbilical vein endothelial cells, BMSC, etc	Antitumor immunity	T cells	FCM, IHC	89

Abbreviations: DCs, dendritic cells; ELISA, enzyme‐linked immunosorbent assay; FCM, flow cytometry; IHC, immunohistochemistry; IL, interleukin; PCL, poly‐ε‐caprolactone; PDO, polydioxanone; PEOT/PBT, poly(ethylene oxide terephthalate)/poly(butylene terephthalate); PVA, polyvinyl alcohol; PVP, polyvinylpyrrolidone; VLP, virus‐like particles.

### 2.2 Dendritic cells

DCs are found in peripheral lymphoid tissues and are classical APCs responsible for activating specific T‐cell responses and maintaining immune tolerance.^90^ They can secrete cytokines, initiate adaptive and innate immunity, regulate T cell differentiation and activation, present antigens, and consequently suppress tumor development.^91^ Tumor immune invasion occurs when DCs become less mature, decrease in number, or differentiate into immunosuppressive/tolerogenic regulatory forms that inhibit T‐cell activation.^92^

**FIGURE 5:**
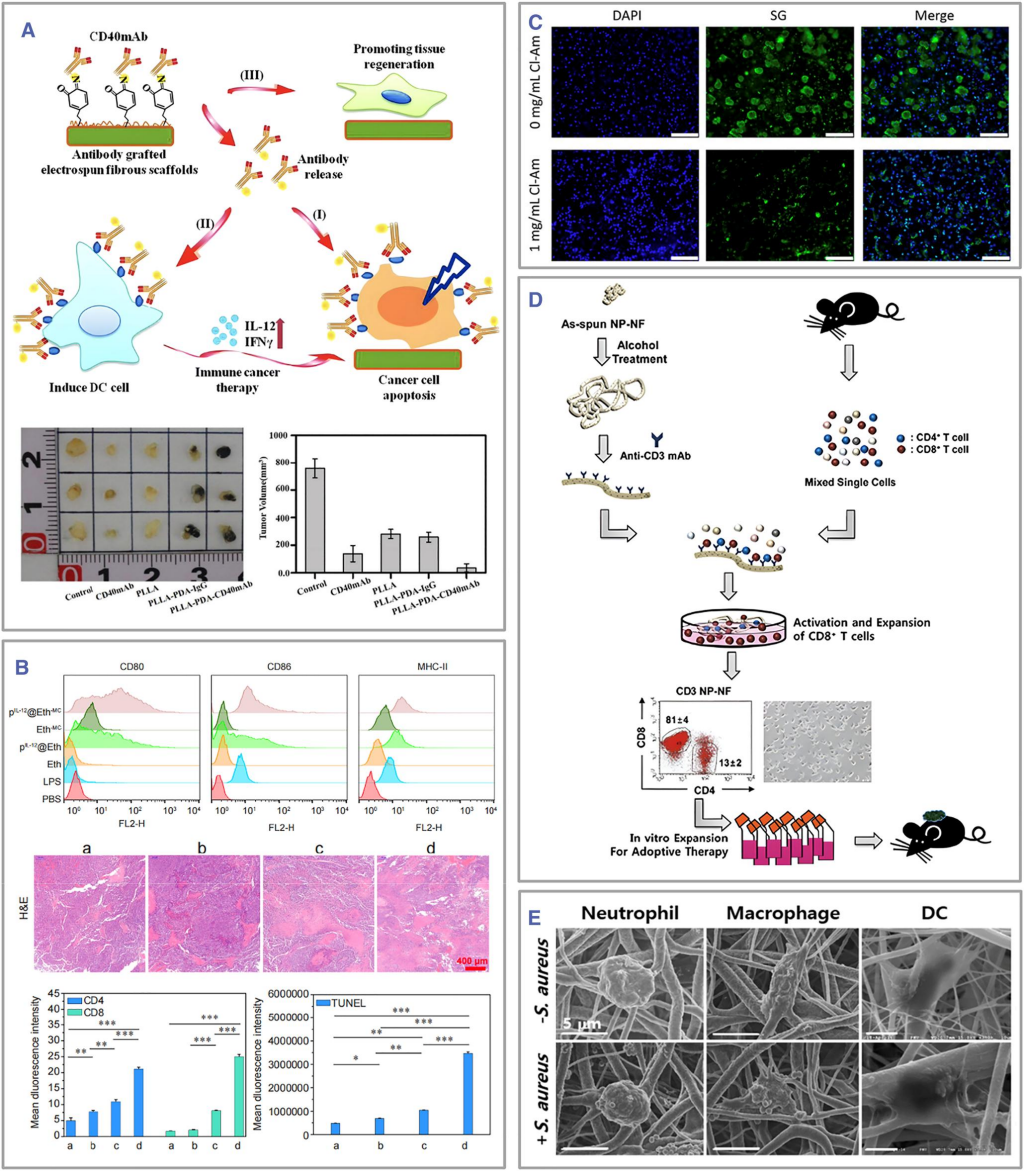
(A) Electrospun fibers surface modified with CD40 mAb can agitate DC cells. Reproduced with permission.^81^ Copyright 2018, The Royal Society of Chemistry. (B) Electrospun fibers capable of releasing IL‐12 can modulate local immunity by influencing both DCs and T cells. *p < 0.05, **p < 0.01 and ***p < 0.001. Reproduced with permission.^82^ Copyright 2021, Chinese Medical Association. (C) Electrospun fibers incorporating CI‐Am possess the capacity to modulate neutrophil aggregation and the generation of NETs. Scale bars: 100 μm. Reproduced under terms of the CC‐BY license.^95^ Copyright 2018, The Authors, published by Frontiers Media S. A. (D) Electrospun fibers grafted with CD3mAb on the surface screen and activate lymphocytes in vitro. Reproduced with permission.^96^ Copyright 2012, American Chemical Society. (E) Electrospinning can mimic in vivo immune responses for research purposes. Reproduced under terms of the CC‐BY license.^93^ Copyright 2021, The Authors, published by MDPI. DC, dendritic cell; IL, interleukin; NETs, neutrophil extracellular traps.

Electrospun fiber‐based immune engineering strategies directed toward DCs in electrospinning frequently materialize via surface modifications or internal incorporation of cytokines conducive to their maturation or recruitment.^93^ For example, DCs were cultured on electrospun fibers, and the surface grafting of CD40 antibodies or IL‐12 plasmids promoted their maturation and enhanced their antitumor immunity.^94^ Liu et al. found that the fiber‐released CD 40 antibodies can activate DCs and secrete IL‐12 and IFN‐γ, restoring immune escape of tumor cells, promoting apoptosis, and increasing antitumor activity (Figure 5A).^81^ Hong et al. designed electrospun fiber scaffolds grafted with microspheres containing IL‐12 to find DC maturation and T‐cell infiltration. In addition, their combination with the programmed cell death protein‐1 monoclonal antibody (aPD‐1) exhibits more substantial antitumor effects than aPD‐1 alone (Figure 5B).^82^

The culmination of research into electrospun fibers reveals a promising frontier in immunotherapy, particularly in the activation and maturation of DCs. The novel application of these fibers for the targeted delivery of cytokines and the functionalization with specific antibodies underscores a significant advancement in stimulating immune responses against tumors, enhancing the efficacy of conventional treatments. Similarly, the application of electrospun fiber technology in tissue repair is bolstered by its capacity to modulate immune responses, with DCs playing a pivotal role. Tailored surface modifications and cytokine incorporation within these fibers have been shown to not only potentiate antitumor effects but also to facilitate tissue repair by enhancing DC function and subsequent T cell activation. This dual functionality illustrates a significant leap in both regenerative medicine and cancer immunotherapy.

### 2.3 Mast cells

Mast cells are essential immune sentinel cells that release large number of cytokines, growth factors, and mitogens, affecting numerous biological processes including immune responses to bacteria and viruses, angiogenesis, wound healing, fibrosis, autoimmunity, metabolic disorders, and cancer.^97^ Therefore, modulating the function of mast cells to regulate inflammation by electrospun fibers presents significant application prospects.

The immune engineering strategies implemented in electrospinning to modulate mast cells are often realized through nuanced modifications in the physicochemical parameters encompassing the composition and topology of the fibers. Abebayehu et al. demonstrated that elevating the polydioxanone (PDO) concentration in electrospun fibers led to reduced secretion of inflammatory cytokines (IL‐6 and TNF) and enhanced secretion of vascular endothelial growth factor (VEGF) by mast cells. Furthermore, fibers' pores more prominent than 4–6 μm triggered diminished release of IL‐6 and TNF by mast cells.^84^

The innovative use of electrospun fiber biomaterials to modulate the mast cell activity offers a promising avenue for enhancing tissue repair and regulating inflammation. Strategic adjustment of fiber composition and structure has been shown to fine‐tune the balance between pro‐inflammatory and regenerative signals. Such nuanced control of mast cell functions via electrospun fibers could lead to more effective therapies for a wide array of conditions ranging from wound healing to the mitigation of fibrotic disorders and cancer.

### 2.4 Neutrophils

Neutrophils are the first recruited cells at the inflammation site. They are produced via the differentiation of progenitor cells in the hematopoietic cords of the bone marrow venous sinuses. Later, they enter the blood circulation and become the predominant leukocytes, with a 5.4‐day lifespan.^25^ Upon infection or tissue injury, pathogen‐associated molecular patterns (PAMPs) and damage‐associated molecular patterns (DAMPs) are recognized by receptors, activating tissue‐resident immune cells to produce inflammatory mediators (e.g., CXCL1 and CXCL2) which can bind to G‐protein‐coupled receptors on neutrophils and activate them.^98^ Activated neutrophils become phagocytic, producing ROS and releasing substances such as neutrophil elastase (NE), cathepsin G, and protease 3, eventually initiating inflammation. Besides, neutrophils can form neutrophil extracellular traps (NETs) to kill the cell (called NETosis) and limit pathogen spread. However, excessive NET production worsens inflammation and host tissue damage beyond the antimicrobial function.^99^

Hence, immune engineering strategies in the context of electrospinning for the regulation of neutrophils are often anticipated to attenuate neutrophil infiltration and NET production, thereby modulating acute‐phase inflammation. Among these, the most widely employed strategy involves incorporating drugs that suppress excessive neutrophil responses. For instance, PDO electrospun fibers loaded with Cl‐amidine significantly reduced the NET production (Figure 5C).^95^

Conclusively, the field of immune engineering through electrospun fibers is advancing toward effectively regulating neutrophil activities to strike a balance in inflammatory responses. By fine‐tuning the interactions between neutrophils and the biomaterials, it is possible to mitigate the overproduction of inflammatory mediators and NETs, thus protecting the tissue from excessive damage. These innovations in material science interfacing with cellular immunology hold significant promise for improving outcomes in tissue repair and reducing the complications associated with chronic inflammation.

### 2.5 Lymphocytes

Lymphocytes, including T and B cells, can be classified into memory, helper, and effector cell subtypes and often function in a specific immune regulation, participating in various pathological processes, such as autoimmune responses and tumor immunity.^100^ B cells can secrete antibodies to promote humoral immunity, and T cells participate in cellular immunity. In the tumor microenvironment, CD8^+^ T cells are considered critical immune cells for targeting cancer cells presenting major histocompatibility complex class I molecules. CD8^+^ T cells must be activated into effector cytotoxic T cells (CTLs) with DCs, NK cells, and CD4^+^ T cells to generate a persistent and effective antitumor response.^101^ Once activated, effector CTLs infiltrate and kill tumor cells by releasing granules or induction of FasL‐mediated apoptosis.

Cancer‐associated fibroblasts, M2 macrophages, and regulatory T cells (Tregs) can also form immune barriers against CD8^+^ T cell‐mediated antitumor responses.^102^ These barriers can lead to dysfunction and exhaustion of CD8^+^ T cells, contributing to adaptive immune resistance in cancer.^103^ In addition, autoimmune T cells mistakenly attack healthy cells and tissues in the body, causing inflammation and damage and eventually contributing to autoimmune disease development.^104^

Given the manifold diversity of lymphocyte subtypes and their intricate role in the initiation and advancement of diverse maladies, the strategies employed in electrospinning‐based immune engineering directed toward the modulation of lymphocytes manifest a multifaceted complexity. Electrospun fibers can either be designed to promote or inhibit lymphocyte infiltration.^87^, ^88^, ^96^ For instance, polyvinylpyrrolidone (PVP) electrospun fibers containing clobetasol‐17‐propionate suppressed the activation of autoimmune T cells, thus showing potential in treating oral lichen planus, an autoimmune disorder.^88^ Fiber surface grafting with CD3 monoclonal antibodies allows for the initial screening and activation of CD8^+^CD3^+^ T cells in vitro (Figure 5D).^96^

### 2.6 NK cells

NK cells are unique members of the immune system that can kill specific cells such as tumor cells and those infected by pathogens, thereby limiting the progression of tumors and the spread of infection without the need for prior sensitization.^105^ Unlike T cells, NK cells do not require antigen processing and presentation for activation. This makes them an essential focus in cancer therapy due to their simple activation cues. Scientists are currently developing and enhancing NK cell‐based cancer immunotherapy to increase its ability to fight tumors. Clinical trials are underway to investigate the use of engineered induced pluripotent stem cells (iPSC)‐NK cells, and other methods of “priming” NK cells in vitro or in vivo are also being explored to maximize their antitumor functions.^106^

However, within the domain of electrospinning immune engineering, provoking the activation of NK cells to unleash their augmented capabilities in annihilating tumors seems to present persistent challenges. Collagen‐containing electrospun fibers may, however, inhibit the functioning of NK cells. In a study, Smith created electrospun fibers with varying ratios by combining PDO and lyophilized type I collagen (CI). The impact of these PDO‐CI blend fibers with different component ratios on the cytotoxicity of NK cells against YAC‐1 tumor cells was evaluated in vitro. It was observed that exposure to all the blend fibers resulted in statistically significant suppression of NK cell activity compared to the control group, with no statistical differences observed among the different mixtures.^107^ In another study conducted by the same research team, PDO and elastin were mixed in various ratios and then electrospun into the mixture to get blended fibers. The NK cell cytotoxicity experiment yielded similar outcomes. Upon co‐culturing with PDO‐elastin blend fibers, a statistically significant reduction in the tumor‐killing capacity of NK cells was observed.^108^

### 2.7 Tissue cells

In addition, tissue cells such as epidermal cells, fibroblasts, and endothelial cells are also involved in immunoregulation via cytokine secretion.^109^ The manipulative tissue cells in the development of inflammation broadly vary in different diseases. Adapting the electrospinning setup to focus on specific tissue cells in specific situations enables immune modulation, presenting new challenges for electrospinning‐based immune engineering. In a study by Danti, poly(ethylene oxide terephthalate)/poly(butylene terephthalate) (PEOT/PBT) electrospun fibers modified with chitin nanofibrils (CNs) on the surface can decrease the secretion of inflammatory factors of human dermal keratinocytes, such as IL‐1 and IL‐8.^110^

Due to the ECM‐like structural properties of electrospun fibers, in addition to regulating inflammation and repair in the body, immune cells and tissue cells can be cultured in vitro to mimic the complex in vivo immune environment.^89^, ^93^, ^94^ This encompasses applications in immune engineering based on electrospun fibers. For example, Lee et al. designed a 3D in vitro PCL electrospun fiber infection model, with *Staphylococcus aureus* cultured on the lower side and neutrophils, macrophages, and DCs on the upper side, to mimic the in vivo inflammatory response to bacterial infection (Figure 5E).^93^ The immune cells cultured on the upper layer migrated to the lower layer containing bacteria. DCs migrated to neutrophils cultured with bacteria in the lower layer and then engulfed them. Additionally, phagocytes from the upper layer move toward bacteria‐infected MLE‐12 lung epithelial cells in the lower layer. *Staphylococcus aureus*‐infected MLE‐12 cells caused the secretion of TNF‐α and IL‐1α in 3D culture conditions but not in 2D culture conditions. Thus, the PCL‐based 3D culture system, including phagocytes and bacteria, mimics the inflammatory response to microbes in vivo and can be used for the biomimetic study of various microbe infections.

## 3 ELECTROSPUN FIBER‐BASED IMMUNE ENGINEERING APPROACHES

Electrospinning in immune engineering facilitates the regulation of various immune cells, and the fundamental rationale behind designing these electrospun fibers can be categorized into four overarching principles: (i) surface modification, (ii) drug loading, (iii) physicochemical parameters, and (iv) biological grafting. These four strategies can be employed individually or in combination to exert immunoregulative activities.

### 3.1 Surface modification

Surface modification of electrospinning through agents like anti‐inflammatory substances, and altering immunomodulatory functionality constitutes a foremost design strategy in electrospun fiber‐based immunological engineering. Adsorbed proteins and adherent cells on the surface of electrospun fibers initiate subsequent inflammatory responses. The fiber surface modification, such as chemical modification, peptide self‐assembly,^111^ and electrospinning spray techniques,^86^ can result in a functional coating layer (Table 3). Most of these modifications mitigate electrospun fibers' immunogenicity while augmenting their biocompatibility, thus directing local immune responses toward an anti‐inflammatory trajectory. In addition, antibodies can be modified onto the fiber surface to screen and activate specific cell populations^96^ or act as a platform for rapid in vitro immunoassays.^114^, ^115^

**TABLE 3:** Applications of electrospun fibers regulate immune cells via surface modification.

Surface modification substance	Polymer type	Surface modification	Application site/disease	Regulation of immune cells	Biological effects produced	Refs
Anti‐inflammatory substances	PCL	Peptide RADA16‐1 and rabbit recombinant Col4	Peripheral nerve repair	Macrophages	Promote macrophage infiltration and increase the proportion of M2	112
	PLA	IL‐10	Skin wound repair	Macrophages	Promote macrophage M2 subtype transition and reduce inflammation	78
	PEOT/PBT	Electrospray technique, CNs	Tympanitis	Epidermal cells	Enhance the natural immune response of keratinocytes	86
Substances altering biocompatibility	PDO	Fibronectin	Revascularization	Mast cells	Mediate mast cell activation	84
	PCL	Peptides	Inflammation	Neutrophils	Attract adhesion of hMSCs, excite neutrophils, and mimic the in vivo environment	80
	PCL	Collagen 1	Tumors	DCs	More uniform distribution of DCs	94
	PEUU	Heparin	Vascular replacement	Leukocytes	Reduce immune cell and platelet aggregation	61
Plasmid‐containing microspheres/liposomes	PLA	Cationic liposomes containing IL‐4 plasmids	Peripheral nerve repair	Macrophages	Promote M2 macrophage polarization	74
	PVP	Self‐assembled microspheres containing IL‐12 plasmids	Subcutaneous tumors	DCs	Increase DC interaction with PD1	82
Antigens/antibodies	PLLA	Polydopamine and CD40mAb	Breast cancer	DCs	Promote DC maturation	113
	PS/PSMA	CD3 monoclonal antibody	Tumors	T Cells	Excite T cells and promote differentiation to CD8^+^ T	96
	PS	MERS‐CoV antigen	Antibody testing	/	Rapid testing of MERS‐CoV antibodies	114

Abbreviations: CNs, chitin nanofibrils; COL, collagen; DCs, dendritic cells; hMSCs, human mesenchymal stem cells; IL, interleukin; MERS‐CoV, Middle East Respiratory Syndrome coronavirus; PCL, poly‐ε‐caprolactone; PDO, polydioxanone; PEOT/PBT, poly(ethylene oxide terephthalate)/poly(butylene terephthalate); PEUU, low‐initial‐modulus poly(esterurethane)urea; PLA, poly‐L‐lactide; PS, polystyrene; PSMA, poly(styrene‐co‐maleic anhydride); PVP. polyvinylpyrrolidone.

#### 3.1.1 Anti‐inflammatory agents

As the electrospun fibers interact directly with the internal environment, direct release of anti‐inflammatory substances grafted onto their surface into the surrounding milieu can be achieved. ECM analogs, anti‐inflammatory cytokines like IL‐10, and other anti‐inflammatory compounds are excellent candidates for electrospun surface modification.^109^

**FIGURE 6:**
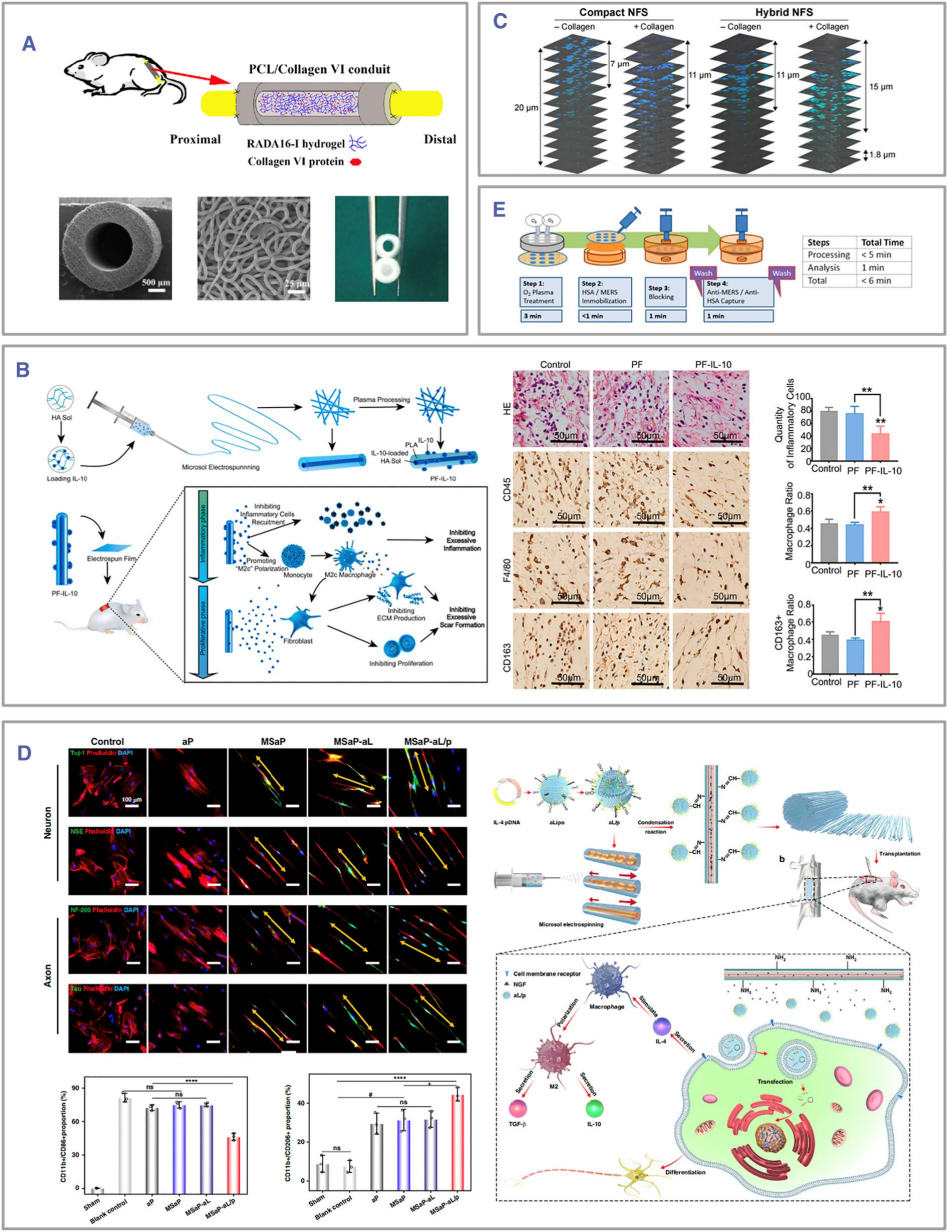
(A) The surface of electrospun fibers can be modified with anti‐inflammatory agents such as collagen 4 for better immune modulation. Reproduced with permission.^112^ Copyright 2017, John Wiley and Sons. (B) The scheme illustrates the structure and immunomodulatory mechanisms of the IL‐10 “inner‐out” electrospun fibers. *p < 0.05, **p < 0.01. Reproduced with permission.^78^ Copyright 2021, The Authors, published by Elsevier. (C) Conducting surface modifications on electrospun fibers or altering fiber topology can transform the material's biocompatibility. Reproduced with permission.^94^ Copyright 2016, The Authors, published by Dove Medical Press Limited. (D) The electrospun fibers surface‐modified with IL‐4 plasmid liposomes facilitate the delivery of the IL‐4 gene, thereby orchestrating an immune modulation and fostering regeneration. *p < 0.05, ^#^ *p* < 0.001 and ****p < 0.0001. Reproduced under terms of the CC‐BY license.^74^ Copyright 2020, The Authors, published by Springer Nature. (E) The modification of electrospun fibers with antibodies allows for antigen detection. Reproduced with permission.^114^ Copyright 2019, The Authors, published by Elsevier. IL, interleukin.

Type IV collagen is reported to have neuroprotective effects and can promote macrophage migration and polarization.^116^ Lv et al. fabricated a PCL electrospun fiber conduit with an average wall thickness of 540 μm and an inner diameter of 1.5 mm for sciatic nerve regeneration. Then, a self‐assembled peptide RADA16‐I hydrogel containing collagen VI protein was injected into the conduit (Figure 6A). Studies conducted in vitro have revealed that the PCL/collagen VI conduit could induce macrophages to polarize toward the M2 phenotype. In vivo experiments were conducted on rats with a 15‐mm‐long defect in the sciatic nerve. Rats were divided into three groups: a PCL/collagen VI conduit group, a PCL conduit group, and an autograft group. The findings demonstrated that the PCL/collagen VI conduit group exhibited enhanced recruitment of macrophages and M2 polarization. Consequently, this group achieved comparable axonal regeneration and neurological functional recovery as the autograft group.^112^

The M2c subtype secretes regulatory factors endowed with anti‐inflammatory and anti‐fibrotic attributes. In the acute inflammatory phase, low levels of IL‐10 inhibit immune cell recruitment, thereby fostering M2c macrophage polarization and consequently inflammatory suppression. On the contrary, elevated concentrations of IL‐10 will result in excessive ECM deposits by fibroblasts during the regenerative phase.^117^ Chen et al. devised a core‐shell electrospun fiber structure to facilitate a sustained release of IL‐10 throughout the regenerative process.^78^ The PLA shell was modified with a lower concentration of IL‐10, which could be quickly released within the initial 5 days after implantation. The core layer, however, was loaded with a higher concentration of IL‐10, manifesting its effects between days six and 15 post‐implantation. The investigation revealed that this uniquely designed electrospun fiber enabled the sequential delivery of IL‐10, inhibiting inflammatory cell clustering, promoting M2c polarization, and promoting tissue regeneration while preventing scar formation (Figure 6B).

PEOT/PBT is a recognized biodegradable polymer complex with excellent performance in otitis media, and CNs are considered to have anti‐inflammatory effects. Danti et al. used the electrospray technique to treat otitis media by coating PEOT/PBT electrospun fibers with CNs from three sources.^86^ CN coating could reduce the production of proinflammatory factors within 24 h and enhance the innate immune response, significantly upregulating the antimicrobial peptides of human beta‐defensin 2 and TGF‐β.

#### 3.1.2 Surface modification to enhance biocompatibility

Electrospun fibers play an excellent platform in cell adhesion, proliferation, and even differentiation. However, the inert materials used for electrospinning pose a significant compatibility issue, limiting the application scope of electrospun fibers. To address this problem, researchers have tried a great deal of strategies through the decades. Surface modification is the most commonly used approach for improving fiber biocompatibility. Proteins, peptides, and drugs are the most popular choices to improve fiber compatibility by changing the hydrophilicity of or increasing cell adhesion and growth, thereby achieving regenerative immune regulation.

Abebayehu et al. modified the surface of PDO electrospun fibers with fibronectin to promote mast cell adhesion and investigate electrospun fibers' role in mast cells' inflammatory and angiogenic responses.^84^ Similarly, Blum et al. modified the electrospun PCL fiber surface with a peptide RGDS‐motif derived from fibronectin in the ECM to promote human mesenchymal stem cells (hMSCs) adhesion. Besides RGDS‐motif, the study utilized a customized peptide sequence with a specific human neutrophil elastase (HNE) cleavage site (AAPV‐motif) covalently immobilized onto PCL fibers. The study investigated the hMSC adhesion on fibers to assess the cleavage capacity of HNE. Meshes modified with CGGGAAPVGGRGDS peptide were investigated regarding hMSC adhesion after HNE cleavage at the C‐terminus of the AAPV sequence. The study aimed to protect the RGDS‐containing peptide sequence of bioactivated PCL fibers from being cleaved by HNE using the aldehyde‐containing hyaluronic acid (proxHA) hydrogel.^80^ In a similar study, Kim et al. used collagen I for the surface coating of electrospun fibers, and their results showed enhanced fiber biocompatibility indicated by increased cell infiltration, adhesion, and proliferation. Confocal microscopy images revealed that the incorporation of collagen elicits profound infiltration of CT26 cancer cells into the dense and hybrid nanofibrous scaffolds within a mere 4 h of cell culturing (Figure 6C).^94^ Zhu and his colleagues constructed surface‐modified electrospun conduits with poly(ethylene glycol) (PEG) and heparin for small‐diameter vessel replacements.^61^ The modified conduit displayed increased biocompatibility and water solubility, promoting the adhesion, proliferation, and migration of human umbilical vein endothelial cells (HUVECs) in vitro while reducing platelet aggregation in vivo.

#### 3.1.3 Surface modification with plasmid‐containing microspheres/liposomes

The surface modification with microspheres or liposomes containing plasmids allows the plasmids to directly enter and be expressed in the target cells surrounding the electrospun fibers, significantly improving the gene transfection capability of electrospun fibers.

Xi et al. designed a sophisticated core‐shell electrospun fiber architecture for peripheral nerve repair.^74^ The fiber's surface was modified with IL‐4 plasmids encapsulated within pH‐sensitive cationic liposomes while internally loaded with nerve growth factors (NGF). After implantation, the meticulously engineered IL‐4 plasmids efficiently permeated the neighboring cells, thereby augmenting the concentration of IL‐4 within the local milieu and facilitating the desired M2 polarization of macrophages. Concurrently, the gradual release of NGF from within the fiber nurtured the nerve cells and fostered the elongation of axons (Figure 6D).

Evidence suggests that the antitumor efficacy of the monoclonal antibody against aPD‐1 depends on the expression of IL‐12 by DCs, which are abundant in cutaneous tissue. Therefore, Hong et al. designed mannosylated chitosan (MC)‐modified ethosomes (Eth‐MC) containing IL‐12 plasmids and mixed the pIL‐12@Eth‐MC with PVP solution, from which microspheres were prepared via the electrospray technique; these were then sprayed onto the surface of electrospun fibroin‐polyvinyl alcohol nanofibers to obtain a PVP‐pIL‐12@Eth‐MC/fibroin‐polyvinyl alcohol composite nanofiber patch (Figure 5B).^82^

#### 3.1.4 Surface modification with antigens/antibodies

Modifying electrospun fibers with antibodies allows electrospun fibers to target acting cells. For example, Liu et al. designed PLLA fibers with grafted murine anti‐human CD40 antibodies (CD40mAb) and polydopamine on the surface to promote DC activation and increase antitumor immunity. CD40, a type I membrane glycoprotein, is typically expressed in B cells and DCs. However, it is also highly expressed in several solid tumors. Combining CD40mAb with TLR3 ligand can efficiently restore DC‐mediated immunity and disrupt tumor suppression. In vitro experiments demonstrated the multifunctionality of the PLLA‐PDA‐CD40mAb scaffold. It directly induced apoptosis in cancer cells and provides physical support to promote cell proliferation. In animal experiments, severe combined immunodeficiency mice were employed to evaluate the therapeutic effect of PLLA‐PDA‐CD40mAb scaffolds on tumor cell apoptosis induction. Compared to the PLLA‐PDA group and PLLA‐PDA‐IgG group, the PLLA‐PDA‐CD40mAb scaffolds significantly inhibited tumor growth and extend the survival time of the mice (Figure 5A).^81^ In another study, Kim et al. designed electrospun fibers containing magnetic nanoparticles grafted with CD3 monoclonal antibodies on the surface to perform the initial screening and activation of CD8^+^CD3^+^ T cells in vitro (Figure 5D).^96^

In addition, grafting antigens on electrospun fibers can be used for antibody testing. Hoy et al. grafted the Middle East Respiratory Syndrome coronavirus (MERS‐CoV) antigens onto electrospun polystyrene (ESPS) membranes and performed color development via western blotting to rapidly detect whether a patient was infected with the virus. The device can be entirely operated within 5 min. The first step was to immobilize the capture molecule on the ESPS membranes. After immobilization, the subsequent step entails surface blocking using skim milk. This was accomplished by directly administering the skim milk‐blocking solution into the cassette via a syringe, allowing it to remain for 1 min before being drawn out through the outlet. The third step involved the capture of the antibody using Fluorescein Isothiocyanate, a fluorescent dye commonly employed in biological research for antibody labeling and detection. For MERS, the cassette was filled with 1 mL of FITC‐conjugated anti‐MERS solution, held for 1 min, and then flushed out. Furthermore, washing was performed with PBS‐T, where 1 mL volumes were flushed through the cassette three times after both the blocking and FITC‐conjugated antibody steps (Figure 6E).^114^ Falcucci et al. designed an in vitro assay device for human papillomavirus oncogenic proteins E7 protein testing, taking advantage of the ability of electrospun fibers to effectively adsorb proteins due to their high surface‐area‐to‐volume ratio when used for enzyme‐linked immunosorbent assay tests. The device, known as ePCL (electrospun PCL microfibrous layers), was developed with modest antigen content and boasts exceptional attributes surpassing conventional polystyrene plates. It amplifies the detection signal of serum antibodies by 5–6 folds while reducing manufacturing expenses.^115^

### 3.2 Drug loading

Apart from the surface modification, constructing electrospun fibers loaded with bioactive components is also feasible. These loaded agents can be evenly dispersed throughout the fibers and released in a controlled and stable manner.^40^ In cases requiring higher drug stability, degradation resistance, or a gradual release pattern, core‐shell or inner‐outer architectures can protect the drug from the surrounding milieu, maximizing its in vivo delivery efficiency.^74^, ^78^ Historically, commonly loaded drugs can be divided into immunoregulative or biologically active drugs.

#### 3.2.1 Immunoregulative drug loading

By incorporating immunoregulative components into electrospun fibers, one can effectively extend the releasing duration, exerting a continuous immunoregulative impact on the surrounding microenvironment and facilitating tissue regeneration.^118^, ^119^

**FIGURE 7:**
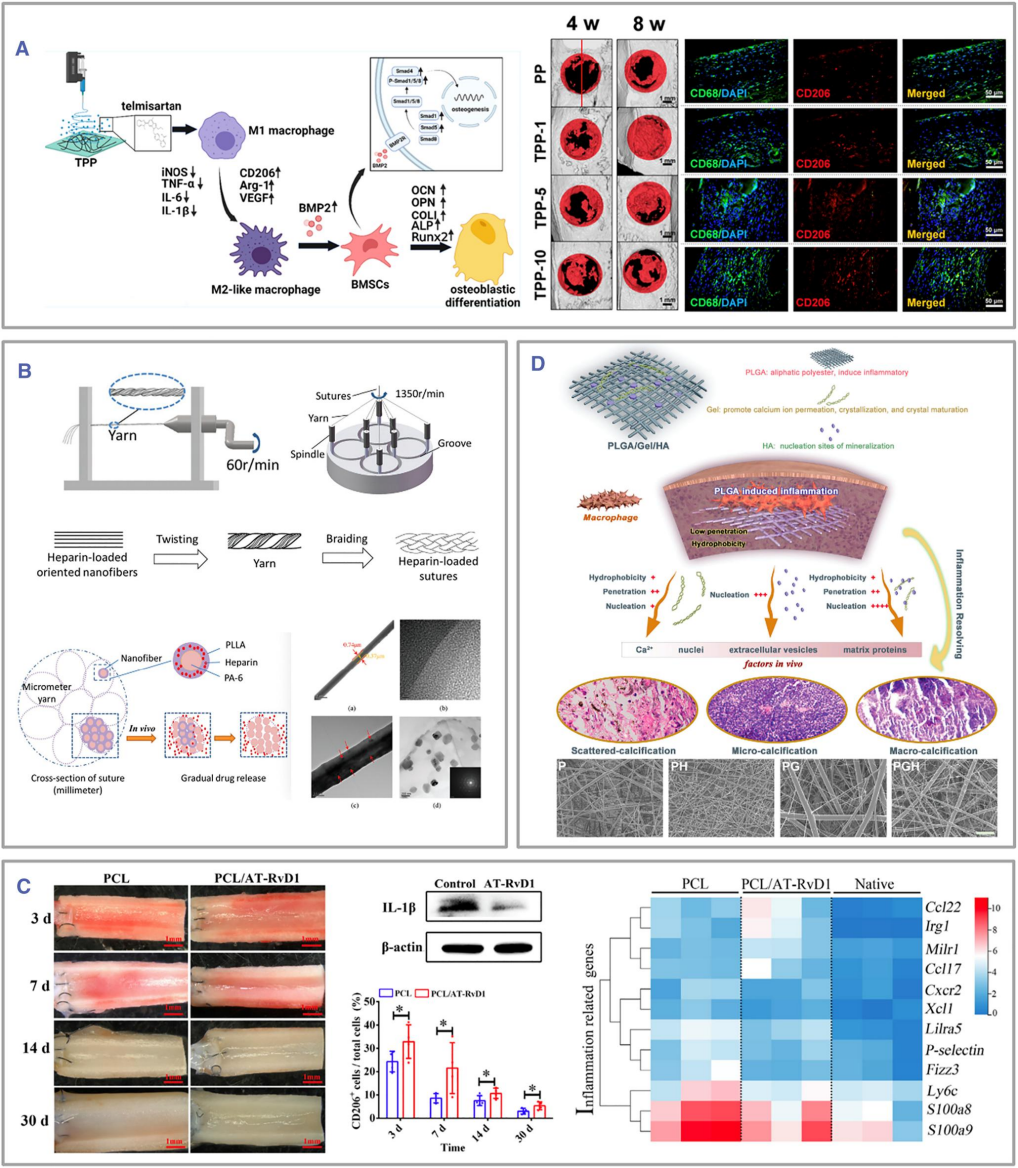
(A) Electrospun fibers loaded with immunoregulative drugs, such as telmisartan, can act as immune modulators. Reproduced with permission.^121^ Copyright 2022, American Chemical Society. (B) Schematic illustration depicting the structure of electrospun sutures laden with heparin. Reproduced with permission.^122^ Copyright 2018, John Wiley and Sons. (C) Electrospun fibers have the ability to encapsulate small molecular immunomodulatory proteins, such as AT‐RvD1, thereby enhancing their efficacy. *p < 0.05. Reproduced with permission.^76^ Copyright 2019, Elsevier. (D) Electrospun fibers could load biologically active natural compounds. Reproduced with permission.^123^ Copyright 2021, John Wiley and Sons.

Oral lichen planus is a T‐cell‐mediated autoimmune disorder that can be treated with topical corticosteroid mouthwashes or sprays.^120^ Given this, Said et al. fabricated glucocorticoid clobetasol‐17‐propionate‐loaded PVP electrospun fibers to extend the localized immunoregulative effect of corticosteroids to suppress T‐cell activation and IL‐2 production, thus aiding in successful disease control.^88^ In another study, Wu et al. successfully constructed telmisartan‐loaded PCL/PVP electrospun fibers, which displayed in vitro anti‐inflammatory bioactivity on lipopolysaccharide‐induced M1 macrophages by polarizing them to an M2‐like phenotype (Figure 7A). In addition, the bone morphogenetic protein‐2 (BMP2)‐Smad signaling pathway plays a crucial role in the interaction between mesenchymal stem cells (MSCs) and macrophages. Based on experimental results, using fibers loaded with telmisartan enhances this interaction, exhibiting pro‐osteogenic properties on bone marrow‐derived mesenchymal stem cells (BMSCs).^121^ Additionally, Ye et al. designed a core‐shell‐structured suture for acute tendon rupture surgery. In their construction, the polyamide (PA)‐6 (PA‐6) with excellent mechanical strength was designed as the core, and while heparin‐loaded PLLA as the shell, the bilayer fiber structure was twisted into a rope for in vivo tissue suturing^122^; compared with non‐heparin‐loaded suture group and commercial suture group, the high heparin concentration leads to milder inflammation and faster healing when applicated in vivo, indicating promising tissue repairing capability of this electrospun suture (Figure 7B). Jiang et al. constructed a complex scaffold by integrating the 1,25‐dihydroxy vitamin D3 (1,25(OH)2D3) into PCL electrospun fiber to induce the expression of the human antimicrobial peptide CAP18/LL37 (hCAP18/LL37) gene in local monocytes and keratinocytes of the incision, thereby achieving postoperative wound resistance to bacterial infection.^85^ Deferoxamine (DFO) is an iron chelator that stabilizes hypoxia‐inducible factor‐1 alpha (HIF‐1α) to enhance angiogenesis, inhibit ROS formation, relieve inflammation, and promote tissue regeneration.^124^ Considering this, Dong et al. fabricated aligned electrospun fibers loading with P(MMD‐co‐LA) to facilitate nerve regeneration.^125^ In vitro experiments demonstrated that the scaffold exhibits sustained release of DFO, thereby enhancing the migration and tube formation of HUVECs and upregulating the expression of angiogenesis‐related genes. Additionally, the oriented nanofibers within the scaffold provide physical cues that effectively regulate macrophage polarization and suppress the expression of inflammatory factors. The scaffold exhibited remarkable regenerative capabilities when applied in a rat model of peripheral nerve damage. The group treated with this scaffold demonstrated elevated levels of M2 polarization accompanied by a reduced inflammatory response in the injured nerve. Furthermore, the in situ release of DFO stimulated the upregulation of HIF1‐α and its target gene, VEGF, thereby promoting revascularization and enhancing nerve regeneration at the defect site.

Moreover, electrospun fibers can encapsulate small‐molecule proteins, including cytokines and growth factors, which are susceptible to enzymatic degradation or hold potential immunogenicity.

IL‐4, a cytokine that stimulates macrophage polarization toward the M2 phenotype, possesses strong immunogenicity and is quickly metabolized in the bloodstream. To address this, Ziemba et al. first attempted to stabilize IL‐4 with bovine serum albumin and encapsulated it within PLLA fiber to decrease overall immunogenicity. In vitro results showed augmented elongation of peritoneal macrophages derived from C57BL/6 mice, increased RNA expression of the anti‐inflammatory marker arginase‐1 (Arg‐1), elevated IL‐10/IL‐12 p40 RNA ratio, and reduced protein expression of proinflammatory markers (IL‐12 p40). These findings collectively demonstrated that electrospinning could promote the polarization of macrophages toward an M2 phenotype, thereby exerting a modulating influence on local immunity.^111^ Similarly, Shi et al. designed PCL small vessel grafts loaded with aspirin‐triggered resolvin D1 to modulate inflammation and induce endothelialization to promote vascular and muscle regeneration (Figure 7C).^76^ In neutrophil‐induced NETosis, the enzyme peptidyl arginase deiminase 4 (PAD4) catalyzes the conversion of histone H3 to citrullinated histone, leading to chromatin decondensation, cellular death, and tissue fibrosis.^126^ Fetz et al. loaded the PAD4 inhibitor Cl‐amidine (Cl‐Am) into PDO electrospun fibers, finding that incorporating Cl‐Am remarkably reduced neutrophil infiltration and the generation of NETs and NETois (Figure 5C).^99^

#### 3.2.2 Biologically active compound loading

Biologically active natural compounds are highly valued for their therapeutic properties, including their ability to fight against microbes, reduce inflammation, and promote tissue regeneration.^127^ These compounds can also be incorporated into electrospun fibers to enhance their therapeutic effectiveness.^128^

Coumarin is a naturally occurring oxygenated bioactive molecule. Zinc oxide possesses effective antimicrobial and bio‐adaptive properties. Meanwhile, magnesium (Mg) exhibits mechanical properties similar to bones and stimulates the transformation of macrophages to osteoclasts. However, Mg degrades quickly in vivo. Negrescu and colleagues combined the abovementioned substances with PCL to create electrospun fibers. Their research findings indicated that this combination significantly reduced inflammation and promoted macrophages to differentiate into osteoclasts.^129^ García‐Salinas and colleagues combined natural compounds from essential oils, including thymol and tyrosol, with PCL to aid in the healing of skin wounds^130^; such compounds can inhibit the NF‐κB pathway and the production of TNF‐α, IL‐1b, and IL‐6, thereby exerting antibacterial and anti‐inflammatory effects. Chitosan stands out as the sole natural alkaline positively charged polysaccharide with impressive biocompatibility, biodegradability, and antibacterial and anti‐inflammatory properties.^131^ Castro and colleagues conducted a study combining chitosan and oxidized carbon nano‐onions with PVA to treat skin wounds. The researchers discovered that this mixture helped extend the material's life and reduce the inflammation caused by the implanted material.^83^ In another similar study, Smith and colleagues successfully blended various ratios of elastin, fetal bovine dermal collagen, and PCL, constructing electrospun fibers that can effectively suppress the activity of T and B lymphocytes, NK cells, and macrophages. Their innovative discovery is crucial to advancing immunoregulative biomaterial applications.^107^, ^108^ Moreover, polyester can also be carried by electrospun fibers.^37^ Nun individually blended four types of polyester with PCL in a 1:1 ratio and used electrospinning to create highly biocompatible fibers. Research on the rat dermal defect model shows that incorporating polyester affects the reorganization of granulation tissue. Specifically, electrospun fibers loaded with bismethoxy ethyl‐functionalized (bMoEt) or isopropyl‐functionalized (iPr) compounds lead to more complete wound healing.

It is essential to consider whether natural compounds maintain their original structure and function after loading. Fish collagen that can be easily extracted is known to have good biocompatibility, poses a low risk of disease transmission, and has minimal religious and ethical concerns. As a result, it has been widely used in the regenerative medicine.^132^, ^133^ Hassanbhai and his team mixed collagen from tilapia skin with poly(lactic‐co‐glycolic acid) (PLGA). They discovered that the fibers created through electrospinning could reduce inflammatory responses and fiber encapsulation. Unfortunately, due to the loading process, the collagen lost its original 3D structure and transformed into gelatin.^134^

In addition to their therapeutic uses, combining polymers with natural compounds can accurately recreate disease conditions in the human body. For example, Wang et al. skillfully combined PLGA, gelatin, and hydroxyapatite employing advanced electrospinning techniques to fabricate fibers that can replicate the biophysical milieu of pathological calcification. This groundbreaking approach allowed for an in‐depth exploration of the intricate interplay between calcification and inflammation (Figure 7D).^123^ In this unique model, PLGA mimics the lipid layer of vascular calcification. When it breaks down, it releases acids that cause inflammation in the area. Additionally, it promotes small patches of mineralization and prevents calcium ions from penetrating. On the other hand, gelatin is similar to ECM proteins and can attract calcium ions. It is also soluble in water, which helps to form and mature calcium deposits in injured areas. The model showed that inflammation gradually decreased as pathological calcification progressed. This leads to minimal inflammatory responses in the mature stage of calcification.

### 3.3 Physicochemical parameters

Furthermore, the physicochemical parameters inherent in electrospinning exhibit diverse levels of impact on its capability to regulate immune functions. The physicochemical parameters of electrospun fibers, including their structure, topology, orientation, and morphology, can influence local immune responses and establish a favorable microenvironment that promotes tissue regeneration. Among all the electrospun fiber physicochemical parameters, fiber component, topology, orientation, and morphology are the most widely explored (Table 4).

**TABLE 4:** Applications of electrospun fibers for regulating immune cells by altering physicochemical parameters.

Physicochemical property altered	Fiber component	Other physicochemical property	Application site/disease	Affected immune cells	Biological effects produced	Refs
Fiber component	Fetal bovine ECM collagen, PHB, PCL, silk, PLA, PA	/	Heart attack	Macrophages	PHB and PLA promote M2 polarization best	135
	PCL + PEI	Highly cationic surface	/	Macrophages	Diminished expression of inflammatory factors	136
	PDO, COL	/	/	Neutrophils	Fewer NETs produced in COL group	137
	MD + amino acid	/	Chronic skin wound repair	Macrophages	Anti‐oxygen radicals, promote M2 polarization	138
	SS:SF = 3:7	/	Revascularization	Macrophages	Secrete more relevant cytokines to promote M2 polarization	139
Fiber topology	PCL	Large/small pore size	Tumors	Dendritic cells	DCs in large pore‐size group is more homogeneous and unactivated	94
	PCL	Large pore size, random	Peripheral nerve repair	Macrophages	Less cell infiltration and promote M2 polarization	73
	PDO	Lattice, large pore size	/	Neutrophils	NETs reduction	140
	PDO, COL	Large/small pore size	/	Neutrophils	Less adhesion at 1 day in large pore size group	137
	PLLA	Coarse fibers, large pore size/fine fibers, small pore size	Bone regeneration	Macrophages	Large pore size and coarse fiber diameter groups promote M2 polarization via the PI3K/AKT pathway	77
	PDO	Coarse fibers, large pore size/fine fibers, small pore size	Revascularization	Mast cells	The thicker the fiber and the larger the pore size, the easier the activation of mast cells	84
Fiber orientation	PCL	Radial aligned fibers	/	Macrophages, T cells	Promote M2 polarization and CD4^+^ T differentiation	141
	P(MMD‐co‐LA)	Aligned double‐layer structure with vertical orientation of inner and outer layers	Peripheral nerve regeneration	Macrophages	Promote M2 polarization	125
	PLGA + fish collagen + chitosan	Random/aligned/lattice	Bone regeneration	Macrophages, monocytes	The lattice group is most likely to recruit monocytes and macrophages; the aligned group causes the least inflammation	142
	PCL	Random/aligned	Tendon	Fibroblasts	Inflammation is more pronounced in the aligned fiber group	143
Fiber shape	PCL	3D‐printed vertically/radially oriented electrospun filaments	Diabetic skin wounds	Macrophages	Promote M2 polarization	144
	3D printed PCL + electrospun PLLA	Layer‐by‐layer	Bone regeneration	Macrophages	Promote M2 polarization via PI3K/AKT	77

Abbreviations: ECM, extracellular matrix; MD, maltodextrin; NETs, neutrophil extracellular traps; PCL, poly‐ε‐caprolactone; PDO, polydioxanone; PEI, poly(ethylenimine); PHB, poly(3‐hydroxybutyrate); PLA, poly‐L‐lactide; PLGA, poly(lactic‐co‐glycolic acid); SF, silk fibroin; SS, sericin.

#### 3.3.1 Fiber component

The physicochemical characteristics of electrospun fibers rely heavily on polymers which make up their structural foundation. Biodegradable polymers like PLA, polyglycolic acid, and PLGA are often used for electrospinning, while non‐biodegradable options include PCL,^145^ and degradable and non‐degradable compounds can result in distinct physiological processes. For instance, biodegradable fibers can cause foreign body reactions, developing giant cells and fiber encapsulation. On the other hand, fibers that are not biodegradable are broken down by phagocytic cells like macrophages and monocytes.^125^ Therefore, choosing the suitable component for a specific application is imperative.

Delia et al. utilized multiple polymers including fetal bovine ECM collagen, poly(3‐hydroxybutyrate) (PHB), PCL, silk, PLA, and PA through electrospinning to aid in the restoration of cardiac tissue after a heart attack.^135^ Experimental findings demonstrated that PHB and PCL fibers were most compatible with heart cells, while collagen, PCL, and PHB fibers reduced the adverse remodeling of cardiac tissues. Among the tested materials, only PHB significantly impacted angiogenesis, indicating that it may be the best option for repairing the heart.

**FIGURE 8:**
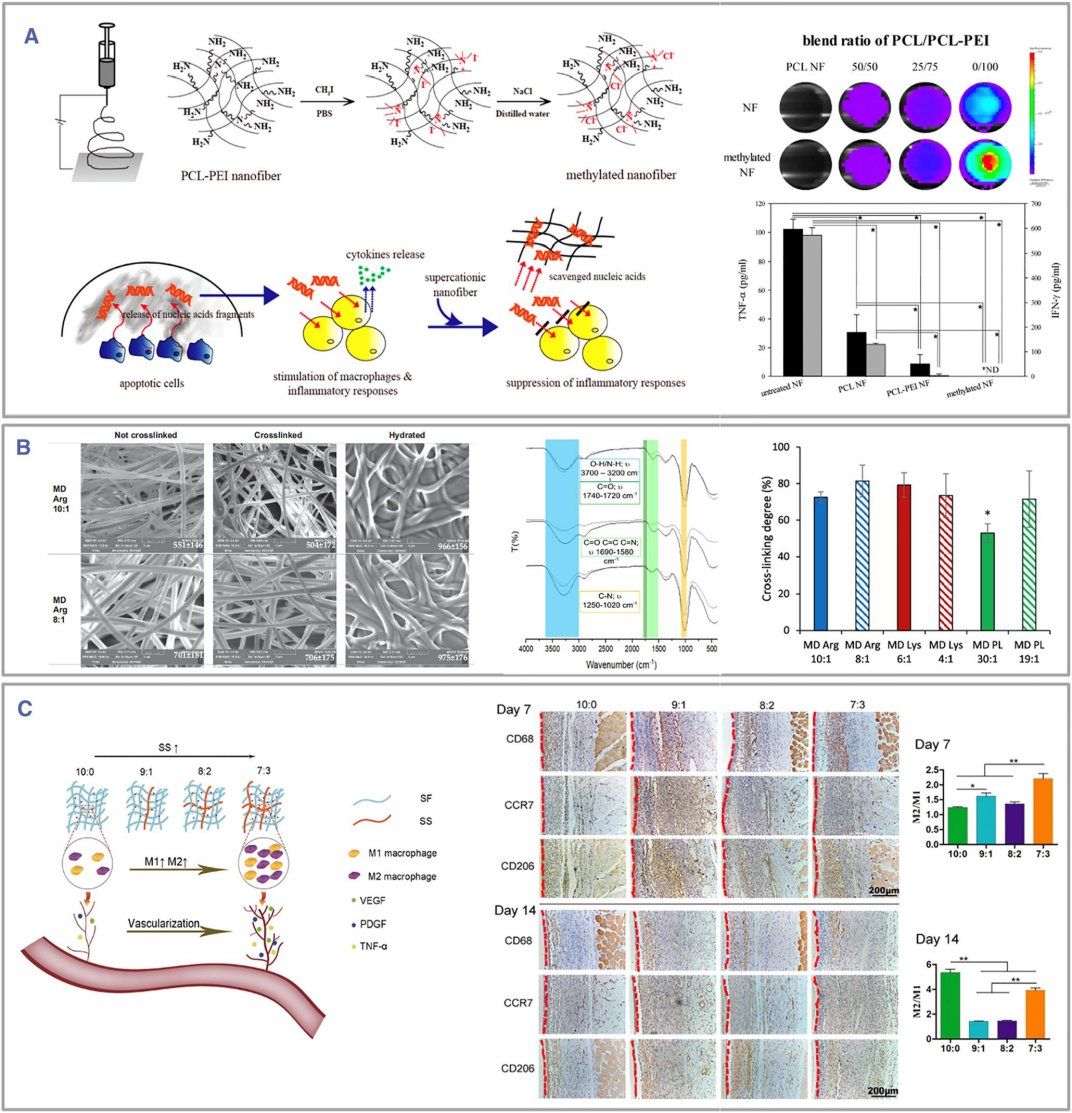
(A) The electrospinning of a blend comprising PCL and PEI in an appropriate ratio enhances their ability to reduce inflammatory reactions efficiently. *p < 0.05. Reproduced with permission.^136^ Copyright 2014, American Chemical Society. (B) Natural macromolecules like MD can interact with amino acids, resulting in electrospun fibers possessing immunomodulatory characteristics. *p < 0.05. Reproduced under terms of the CC‐BY license.^138^ Copyright 2021, The Authors, published by Elsevier. (C) Electrospinning with different SS: SF ratio could induce M2 polarization to modulate the immune response. *p < 0.05 and **p < 0.01. Reproduced with permission.^139^ Copyright 2020, American Chemical Society. PCL, poly‐ε‐caprolactone; PEI, poly(ethylenimine); SF, silk fibroin; SS, sericin.

Furthermore, combining different polymers with various characteristics in appropriate proportion is possible, enabling electrospinning to enhance their immunomodulatory properties with greater effectiveness. For example, Kang et al. fabricated an electrospun fiber with a highly positive surface by co‐spinning PCL and polyethylenimine. This structure effectively collected negatively charged free oligonucleotides from deceased cells in the ECM. This process prevented their inflammatory stimulation through the TLR pathway and promoted restoring the immune balance in the surrounding area (Figure 8A).^136^

In addition, natural macromolecules like polysaccharides and proteins, which have immunomodulatory properties, can also be considered for electrospinning. For instance, Fetz et al. employed mouse tail collagen for electrospinning and found a significant reduction in NETosis compared to the PDO and blended PDO‐COL templates (PC) groups.^137^ Ruggeri et al. combined maltodextrin with arginine, lysine, or polylysine to create a mixture for spinning, which was then subjected to thermal treatment to cross‐link and undergo the Maillard reaction (Figure 8B). The result produced a large amount of melanoidin with antioxidant properties^138^ which could reduce local inflammation, promote M2 macrophage polarization, and enhance chronic wound healing. The natural properties of silk fibroin (SF) and sericin (SS) have gained considerable attention in the field of regenerative medicine.^146^, ^147^ SS, bearing immunological resemblance to natural silk, could induce polarization of macrophages toward the M1 phenotype, thereby initiating vascular regeneration through the stimulation of VEGF secretion. Conversely, SF demonstrated low immunogenicity and remarkable biocompatibility. Wang and colleagues combined SF and SS in different proportions and found that a blend ratio of 7:3 resulted in electrospun fibers that strongly attracted more macrophages. This led to the secretion of M1 and M2 cytokines with a high M2/M1 ratio and promoted vascular regeneration (Figure 8C).^139^

#### 3.3.2 Fiber topology

The topography of electrospun fibers, particularly the pore size and fiber diameter, can modulate immune cells like macrophages, neutrophils, and mast cells, ultimately impacting the immune response.^38^

**FIGURE 9:**
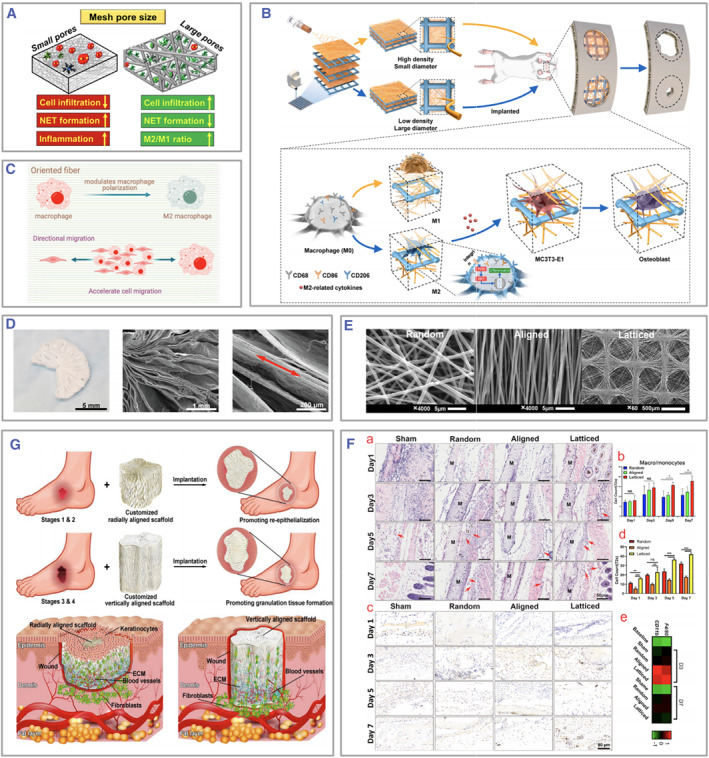
(A) Diverse fiber pore sizes can elicit disparate biological reactions. Reproduced with permission.^148^ Copyright 2022, Elsevier. (B) Scheme of electrospinning combined with 3D printing for skull repair; large pore size promotes macrophage M2 polarization. Reproduced with permission.^77^ Copyright 2021, Elsevier. (C) Oriented fibers could promote macrophage polarization toward M2 and cell migration. Reproduced with permission.^125^ Copyright 2022, Elsevier. (D) A broad view and sem images of radially oriented fibers. Reproduced with permission.^141^ Copyright 2019, Elsevier. (E) SEM images of different orientation electrospun fibers. Reproduced with permission.^142^ Copyright 2021, Elsevier. (F) Divergent fiber orientations elicit varying degrees of immune responses. Reproduced with permission.^142^ Copyright 2021, Elsevier. (G) The morphology of fibers should be customized to suit specific applications, such as diabetic wound dressings designed to conform to the shape of the wound. *p < 0.05, **p < 0.01 and ***p < 0.001. Reproduced with permission.^144^ Copyright 2020, Elsevier.

Research indicates that when the size of pores increases, there is a corresponding increase in cellular infiltration, a higher ratio of M2/M1 macrophages, fewer NETs produced by neutrophils, and less severe inflammation (Figure 9A).^148^ According to Kim and colleagues, using PCL with a larger pore size could result in better infiltration of cells without triggering any immunogenic activation. One benefit was the potential to co‐culture tumor cells and immune cells in a 3D environment in vitro, which could aid in studying antitumor drugs (Figure 6C).^94^ Sarhane's team developed a non‐oriented PCL material with larger pore sizes, resulting in fewer macrophages and an increase in the M2 macrophage ratio at the wound location. Results showed that it was more effective in promoting nerve repair and muscle reinnervation while also limiting the formation of intraneural scars at the repair site compared to AxoGuard, a clinically used 3D tubular structure made of an ECM from the porcine small intestinal submucosa.^73^ Furthermore, the near‐field electrospinning (NFES) technique is a novel approach that decreases the air gap in classic electrospinning to a millimeter scale. King III and Bowlin obtained a lattice‐like PDO fiber with a large pore size via NFES, highlighting that such fiber led to lower levels of neutrophil activation and fewer NETs.^140^

Moreover, the fiber diameter similarly impacts regulating the immune system by increasing the pore size. Fetz et al. utilized electrospinning techniques to fabricate rat tail collagen, PDO, and PC fibers and found that larger fiber diameter resulted in lower NET formation, promoted tissue repair, and reduced fibrosis 7 days after in vivo animal implantation.^137^ In their study, Liu and colleagues discovered that PLLA electrospun fibers that were micron‐sized (with an average diameter of 2.61 μm) were better at promoting macrophage polarization toward M2 than those that were nano‐sized (with an average diameter of 0.52 μm) (Figure 9B).^77^

Changes in pore size and fiber diameter can affect macrophages, neutrophils, and other immune cells such as mast cells. A research team led by Abebayehu conducted a study where they modified the surface of electrospun fibers with fibronectin. This modification enabled mast cells to adhere to the fibers, facilitating further investigations. When comparing fibers made from 60 mg/mL PDO textiles with low aperture to those made from 140 mg/mL PDO textiles with high aperture, it was found that the latter resulted in less secretion of IL‐6 and TNF by mast cells but more secretion of VEGF.^84^ In addition, enlarging the pore size of PDO fibers with a concentration of 60 mg/mL using the air‐flow mandrel method may lower the production of proinflammatory substances such as IL‐6 and TNF compared to the initial fibers.

#### 3.3.3 Fiber orientation

The orientation of fibers can impact how cells, particularly macrophages, adhere to them. The presence or absence of fiber orientations plays a significant role in this process. Aligned fibers tend to induce M2 macrophage polarization and relieve inflammation compared to random fibers (Figure 9C).^125^ Chen et al. used the gas‐foaming technique to design radially oriented fibers and observed that the resulting fibers promoted CD4^+^ T cell differentiation and M2 macrophage polarization (Figure 9D).^141^ In another similar study, Dong et al. constructed aligned and random nanofiber membranes using P(MMD‐co‐LA), noting that the aligned fibers promoted M2 macrophage polarization, reduced inflammation, and enhanced the migration of HUVECs and angiogenesis.^125^ Jin et al. fabricated three types of fibers: unoriented, oriented, and latticed. Their study found that the latticed fibers were the most effective at recruiting monocytes and macrophages, initiating vascularization, and promoting bone regeneration. On the other hand, the oriented fibers caused less inflammation compared with the unoriented (Figure 9E,F).^142^

Furthermore, it has been reported that cells tend to grow in a specific direction on aligned fiber surfaces while they show random growth on surfaces that lack order. As a result, it is feasible to replicate healthy and damaged tendons by using aligned and random PCL fibers, respectively. Given that, Schoenenberger and colleagues conducted a study exploring the molecular mechanisms involved in tendon injury by designing aligned and random PCL fibers and then co‐cultivating them with fibroblasts and macrophages. According to the research, the genes responsible for breaking down the ECM, such as matrix metalloproteinases found in fibroblasts, were more active in random fibers. On the other hand, the genes responsible for synthesis were less active.^143^

#### 3.3.4 Fiber morphology

There is a great deal of variation in the morphology of electrospun fibers. Typically collected from a cylindrical roller, these fibers can spiral into tubes or spread into thin sheets. For example, when deployed for applications such as blood vessels or nerve regeneration, the electrospun fibers were often rolled into tubes to provide optimal coverage for the injured tissue, with the tube diameter easily modifiable based on individual requirements.^76^, ^112^ Conversely, when utilized to treat conditions like pelvic organ prolapse, abdominal wall hernias, or skin wound repair, the electrospun fibers were usually flattened and placed over the affected area.^75^, ^78^, ^149^ In addition, to facilitate tendon suturing surgeries, the electrospun fibers could also be manipulated into suture lines.^122^

What's more, the integration of 3D printing technology has expanded the scope of electrospinning applications. When harnessed in conjunction with 3D printing technology, it becomes feasible to procure custom‐designed 3D electrospun fibers in virtually any shape imaginable. Chen et al. merged the two technologies to create custom perforations that radiate or are perpendicular based on the shape of chronic diabetic wounds, promoting wound healing (Figure 9G).^144^ Similarly, Liu et al. employed a layer‐by‐layer structure using PLLA electrospinning fiber and PCL mesh, obtained through 3D printing, for cranial bone regeneration. The printed layers could provide physical stability, while the electrospun structures could aid in tissue regeneration by mimicking the structure of the ECM (Figure 9B).^77^

### 3.4 Biological grafting

In addition to the three aforementioned strategies, grafting biomimetic substances is a commonly employed design approach in electrospinning. Materials devised through this method are called “living materials.”^150^ Decellularized extracellular matrices derived from pigs and cows are commonly used in medical procedures such as replacing heart valves and repairing abdominal hernias.^151^ Despite their popularity, these decellularized tissues suffer from the dual setbacks of low mechanical strength and vulnerability to degradation, infections, and wound rupture.^152^, ^153^ Biomaterial researchers are currently focusing on living materials, specifically emphasizing constructs based on MSCs.^150^ MSCs secrete numerous mediators that possess anti‐inflammatory and immune‐regulatory properties. These mediators exhibit diverse anti‐inflammatory and immunomodulatory effects.^154^ Meanwhile, MSCs can differentiate into specific cell types needed for tissue regeneration.^155^, ^156^ Unfortunately, these cells have a limited lifespan, hindering their regenerative applications. Electrospinning technology has the potential to mimic ECM structures and has become a promising method for carrying cells and biological tissues. It can make it possible to extend the period of efficacy, which is ideal for therapeutic interventions while allowing researchers to replicate physiological mechanisms in vivo.

#### 3.4.1 Cells/tissues

**FIGURE 10:**
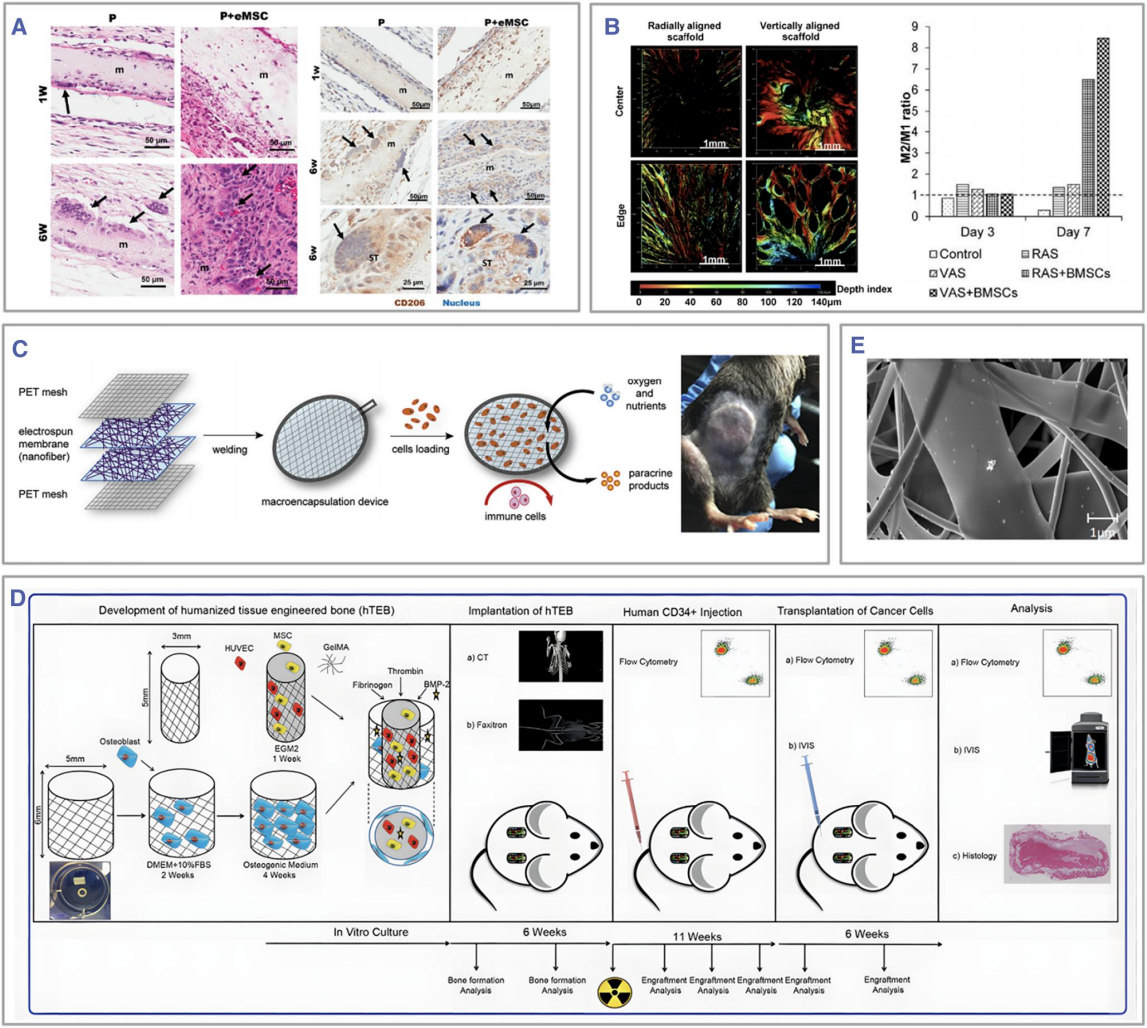
(A) Electrostatically spun fibers can piggyback on eMSCs and cause less inflammation. Reproduced under terms of the CC‐BY license.^75^ Copyright 2020, The Authors, published by Frontiers Media S. A. (B) BMSCs can be cultured on electrospun fibers of various morphologies, promoting M2 polarization of macrophages and acting as immunomodulatory agents. Reproduced with permission.^144^ Copyright 2020, Elsevier. (C) Scheme of employing electrospinning technology to obtain a semi‐permeable immune isolation device. Reproduced with permission.^158^ Copyright 2016, Elsevier. (D) Scheme of the construction of a bone‐like organ with electrospun fibers and associated cells. Reproduced with permission.^89^ Copyright 2018, John Wiley and Sons. (E) Electrospinning provides a platform to encapsulate viral particles. Reproduced under terms of the CC‐BY license.^87^ Copyright 2021, The Authors, published by MDPI. BMSCs, bone marrow‐derived mesenchymal stem cells; eMSCs, endometrial mesenchymal stem cells.

Endometrial mesenchymal stem cells (eMSCs) are commonly acknowledged for their ability to differentiate into various cell types. Studies have shown that injecting eMSCs into damaged pelvic tissue can boost the expression of markers and factors linked to M2, improving inflammation and promoting healing. However, the limited survival time of eMSCs is still a drawback.^157^ To overcome this challenge, Mukherjee et al. developed a Poly(L‐lactide‐co‐caprolactone) electrospun mesh, surface‐grafted with eMSCs, to treat pelvic organ prolapse. Interestingly, they discovered that these meshes continued to possess immune regulatory capabilities following implantation, reducing foreign body giant cells and promoting angiogenesis (Figure 10A).^75^ Similarly, Chen et al. conducted a study where they implanted bone marrow MSCs (BMSCs) onto PCL electrospun fibers to treat chronic diabetic wounds. BMSCs can differentiate into various cells and secrete multiple cytokines to accelerate the wound‐healing response. For 8 weeks or more, the electrospun fiber provided a suitable setting for sustaining the shape and development of stem cells.^144^ It's worth noting that when BMSCs were grafted onto electrospun fibers, it helped prevent the formation of M1‐type macrophages and decreased the production of proinflammatory cytokines like IL‐6 and TNF‐α. Additionally, it promoted the formation of M2‐type macrophages and enhanced the expression of anti‐inflammatory cytokines like IL‐4 and IL‐10 (Refer to Figure 10B).

The cells mentioned earlier, grafted onto electrospun fibers, only grow on the surface and interact directly with the environment around them.^159^ Wang's team studied to protect implanted cells and established an immune‐evading environment by developing a semi‐permeable device using polyurethane electrospun fibers, MSCs cells, and polyethylene terephthalate meshes.^158^ This device allowed for transferring oxygen, nutrients, and cell secretions while monitoring the body's fibrosis or phagocytosis. After being implanted, it was observed that the cells could survive and carry out their intended immunomodulatory function (Figure 10C).

However, the grafted cells or tissues may have limited immunoregulative effects. In the study conducted by Lesage and colleagues, they utilized maternal amniotic fluid obtained from a 17‐week diagnostic amniocentesis test to grow MSCs for the treatment of congenital diaphragmatic hernia. They concluded that PLA electrospun fibers were a suitable culture substrate for AF‐MSCs. However, the presence of AF‐MSCs had minimal impact on macrophage polarization and ECM formation.^160^ Additionally, Maring et al. obtained consent from heart transplant patients with end‐stage dilated cardiomyopathy and extracted the left ventricular myocardium from their transplanted hearts. They then created a hydrogel from the human heart ECM and placed it on top of electrospun fibers made of polyesteretherurethane; however, they found that the fibers elicit only a mild immune response and that the overlay of the human ECM exerts a limited impact.^113^

Apart from therapeutic applications, biological grafting electrospun fibers can also serve as organoids. For instance, Shafiee et al. constructed a bone organoid by co‐culturing preosteoblasts, MSCs, endothelial cells, and hematopoietic cells on the electrospun‐hydrogel hybrid and then implanted them in mice to study the interplay between breast cancer and bone metastasis (Figure 10D).^89^

#### 3.4.2 Microorganisms

Electrospun fibers can serve as a platform for the growth of microorganisms, including bacteria and viruses, in addition to human cells and tissues. Lee and colleagues constructed a 3D in vitro infection model using PCL nanofibers. The model mimicked the inflammatory response to bacterial infection in vivo, with *S. aureus* cultured on the lower surface and neutrophils, phagocytes, and DCs on the upper surface (Figure 5E).^93^ Dowlath et al. designed PVP electrospun membranes containing virus‐like particles derived from the rabbit hemorrhagic disease virus, which was modified to carry antigenic epitopes of the MHC‐I gp100 tumor‐associated antigen (Figure 10E).^87^ Electrospun fibers have a distinct dryness that keeps the virus antigen active. These fibers can act as vaccines by promoting T‐cell activation and interferon production, enhancing antitumor immunity.

## 4 CHALLENGES AND FUTURE OUTLOOK

Generally, biomaterials and the immune system have a complex and inseparable connection. The activation degree of the immune system often determines the outlook for diseases and the destiny of biomaterials. Implanted biomaterials from foreign sources can trigger an immune response from the host.^19^ Well‐designed biomaterials can potentially regulate the immune microenvironment in a local setting. Out of all the different biomaterials available, fibers created through electrospinning technology have shown the most potential for regulating local immunity. This is due to their exceptional biocompatibility, high porosity, high surface‐to‐volume ratio, and stable drug release rates. Although there have been notable advancements in electrospinning‐based immunomodulation research, several challenges still exist.

1. When designing materials, it is essential to consider the immunoregulatory impact of electrospun fibers. To this end, a combination of four strategies—surface modifications, drug loading, physicochemical parameters, and biological grafting—can be implemented in a rational design. Surface modification or internal drug loading is one way to load biologically active substances onto electrospun fibers. It is essential to test and compare both methods to determine which results in the most significant effect with the lowest risk of immunogenic response. The selected method can then be used for subsequent experiments. It is essential to consider the physicochemical parameters and biological grafting when designing for a specific application environment to ensure its intended application.
2. It is necessary to create electrospun fibers that regulate various immunological stages and immune cells. For instance, the ideal fibers utilized to prevent inflammation and promote regeneration should have the ability to inhibit inflammation at multiple stages. This can be achieved by adsorbing and eliminating inflammation‐inducing DAMPs and ROS and hindering the aggregation and activation of neutrophils and other immune cells at the onset of inflammation. It is vital to promote macrophage polarization and promote tissue regeneration at a critical time while avoiding chronic inflammation and fibrosis. Furthermore, electrospun fibers designed to boost antitumor immunity should be able to stimulate DCs and activate related immune cells, including the NKs and CTL cells, simultaneously to unleash more robust antitumor immune effects.
3. The immunoregulatory mechanism of electrospun fibers should be investigated in depth. Most studies on the immunoregulatory function of fibers have focused on determining the quantity and type of macrophages present and the number of immune cells recruited to the implantation site. For instance, it is worth exploring whether electrospun fibers impact the local immune microenvironment by activating a particular signaling pathway or metabolic events.
4. Currently, research on the immune system regulation of electrospun fibers has primarily been carried out on small animals like rats and rabbits, resulting in noteworthy regulatory outcomes. However, clinical translation of electrospun fibers is limited due to the scarcity of research conducted in larger animals or humans. Further large animal research on electrospinning fibers and their immunomodulatory effects will yield more reliable evidence and will speed up the process of clinical translation, ultimately leading to better outcomes for patients.
5. In addition to their application in tissue regeneration, electrospun fibers can be used as a model to mimic the in vivo immune microenvironment. This allows researchers to delve into the intricate workings of the immune system for physiological or pharmacological studies. Currently, the models available are limited and cannot accurately replicate the complex in vivo environment of the immune system, often only including a limited range of immune cells.
6. When combined with other biomaterials, such as 3D‐printed materials, liposomes, and hydrogels, electrospun fibers can achieve more complex immunoregulation and eventually promote tissue regeneration.

## AUTHOR CONTRIBUTIONS

Yiru Xu and Qimanguli Saiding wrote the manuscript. Xue Zhou and Juan Wang helped with the draft. Xinliang Chen and Wenguo Cui provided crucial review and supervised the manuscript. All authors have read and approved the final manuscript.

## CONFLICT OF INTEREST STATEMENT

The authors declare no conflicts of interest.
